# Ribosome biosynthesis and Hedgehog activity are cooperative actionable signaling mechanisms in breast cancer following radiotherapy

**DOI:** 10.1038/s41698-023-00410-y

**Published:** 2023-06-28

**Authors:** Brandon J. Metge, Heba A. Alsheikh, Dongquan Chen, Amr R. Elhamamsy, Dominique C. Hinshaw, Bo-Ruei Chen, Barry P. Sleckman, Rajeev S. Samant, Lalita A. Shevde

**Affiliations:** 1 Department of Pathology, University of Alabama at Birmingham, Birmingham, AL USA; 2 Division of Preventive Medicine, Department of Medicine, University of Alabama at Birmingham, Birmingham, AL USA; 3 O’Neal Comprehensive Cancer Center, University of Alabama at Birmingham, Birmingham, AL USA; 4 Center for Clinical and Translational Sciences, University of Alabama at Birmingham, Birmingham, AL USA; 5 Division of Hematology Oncology, Department of Medicine, University of Alabama at Birmingham, Birmingham, AL USA; 6 Birmingham VA Medical Center, Birmingham, AL USA

**Keywords:** Breast cancer, Metastasis

## Abstract

Hyperactivated ribosome biosynthesis is attributed to a need for elevated protein synthesis that accommodates cell growth and division, and is characterized by nucleomorphometric alterations and increased nucleolar counts. Ribosome biogenesis is challenged when DNA-damaging treatments such as radiotherapy are utilized. Tumor cells that survive radiotherapy form the basis of recurrence, tumor progression, and metastasis. In order to survive and become metabolically revitalized, tumor cells need to reactivate RNA Polymerase I (RNA Pol I) to synthesize ribosomal RNA, an integral component of ribosomes. In this study, we showed that following radiation therapy, tumor cells from breast cancer patients demonstrate activation of a ribosome biosynthesis signature concurrent with enrichment of a signature of Hedgehog (Hh) activity. We hypothesized that GLI1 activates RNA Pol I in response to irradiation and licenses the emergence of a radioresistant tumor population. Our work establishes a novel role for GLI1 in orchestrating RNA Pol I activity in irradiated breast cancer cells. Furthermore, we present evidence that in these irradiated tumor cells, Treacle ribosome biogenesis factor 1 (TCOF1), a nucleolar protein that is important in ribosome biogenesis, facilitates nucleolar translocation of GLI1. Inhibiting Hh activity and RNA Pol I activity disabled the outgrowth of breast cancer cells in the lungs. As such, ribosome biosynthesis and Hh activity present as actionable signaling mechanisms to enhance the effectiveness of radiotherapy.

## Introduction

Ribosome biogenesis is an indispensable cellular process for protein synthesis. The process of ribosome synthesis largely takes place in the nucleolus, with the production of the polycistronic 47S ribosomal RNA (rRNA) precursor (47S Pre-rRNA), which is then processed into 18S, 5.8S, and 28S rRNA. Together with 47 ribosomal proteins, 5S, 5.8S, and 28S rRNA are assembled into the 60S ribosome subunit, while 18S rRNA and 33 ribosomal proteins form the 40S subunit. These 40S and 60S subunits are exported to the cytoplasm to form the mature 80S ribosome. The key rate-limiting step in ribosome genesis is the activity of the multi-subunit enzyme, RNA Polymerase I (RNA Pol I), that is responsible for transcription of 47S rRNA in the nucleolus. As such, nucleoli are critical subnuclear domains in all mammalian cells^1,2^.

The nucleoli form around nucleolar organizer regions (NORs) that encompass ~300–400 ribosomal DNA (rDNA) repeats and are situated on the short arms of the five acrocentric human chromosomes^3^. At the ultrastructural level, the nucleolus is organized into three morphological subdomains. These include the fibrillary center which contains rDNA together with transcription factors such as Upstream binding factor 1 (UBF1) that are essential for rDNA transcription, the dense fibrillar component that houses newly synthesized rRNA molecules for early processing, and the granular component where mature rRNAs are assembled with ribosomal proteins to yield mature ribosome subunits^2^. Given their vital role in protein synthesis, nucleoli are acutely responsive to situations that inflict stress, with responses ranging from morphological and functional changes to molecular responses that can impact cellular homeostasis. Tumor cells are marked by morphological changes in the nucleolus and a higher nucleolar count^2,4^. It is likely that prominent nucleoli seen in tumor cells enable the elevated translation rates needed to fulfill the demand for increased protein synthesis. In breast cancer patients, nucleomorphometric alterations and increased nucleolar counts are associated with reduced breast cancer-specific survival and distant metastasis-free survival^4,5^.

Radiotherapy is commonly employed as an adjuvant treatment modality to limit metastatic spread and achieves favorable outcomes with improvements in both overall and disease-free survival^6^. Radiotherapy inflicts DNA double-strand breaks that disrupt the integrity of rDNA thereby impacting RNA Pol I activity and subsequent rRNA synthesis. It is suggested that the nucleolar DNA damage stress response in actively transcribing nucleoli is rapid and executed by the non-homologous end joining (NHEJ) pathway, while homologous recombination (HR) is invoked to resolve persistent rDNA breaks^7,8^. Our recent work demonstrated that the Hedgehog (Hh) pathway transcription factor Glioma-Associated Oncogene 1 (GLI1) plays an important role in the timely repair of double-strand breaks in rDNA^9^. We hypothesized that GLI1 activates RNA Pol I in response to irradiation and licenses the emergence of a radioresistant tumor population.

While radiotherapy has demonstrated notable success in mitigating breast cancer metastasis, ~34% patients present with local recurrence and/or metastases^10^. Tumor cells that survive the insults of therapy form the basis of recurrence, tumor progression, and metastasis. In order to survive and become metabolically active, tumor cells need to respond to nucleolar DNA damage stress and activate ribosome biogenesis. Here we demonstrate that there is notable activation of a ribosome biosynthesis signature in breast tumors after radiation therapy. This is accompanied by the activation of an epithelial-to-mesenchymal transition (EMT) program that functionally executes invasion, metastasis, and therapy resistance. Inhibiting the activity of RNA Pol I in irradiated tumor cells significantly mitigated pulmonary colonization; furthermore, inhibiting RNA Pol I and Hh/GLI activity enhanced the effectiveness of radiotherapy in subverting tumor growth. As such, recovery of RNA Pol I activity is indispensable for tumor cells to survive and metabolically revitalize after irradiation-inflicted insult. Our work presented here elucidates a signature of Hh/GLI activity in irradiated breast tumors and defines a previously unknown pivotal molecular mechanism that regulates RNA Pol I activation following irradiation. We present novel evidence that in these irradiated tumor cells, Treacle ribosome biogenesis factor 1 (TCOF1), a nucleolar protein that is important in ribosome biogenesis, facilitates nucleolar translocation of GLI1. Cumulatively, this study establishes a novel role for nucleolar GLI1 in orchestrating recovery of RNA Pol I activity, presenting ribosome biosynthesis and Hh activity as actionable signaling mechanisms in irradiated breast cancer cells.

## Results

### Activation of RNA Pol I is critical for the survival of irradiated tumor cells

**Fig. 1:**
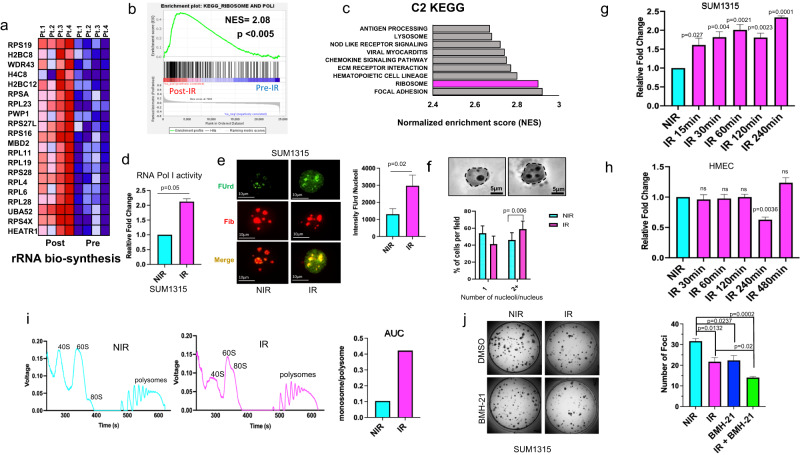
Activation of RNA Pol I is critical for the survival of irradiated tumor cells. **a** Heatmap represents top enriched rRNA biosynthesis signature genes in four matched breast cancer tumors post IR compared to pre-IR. **b** GSEA of rRNA biosynthesis signature (223 genes) examined from tumors of 27 breast cancer patients before IR or 10 days after IR, the graph is showing enrichment of rRNA biosynthesis signature in tumors post IR (NES = 2.08). **c** KEGG top 10 pathways in tumor samples after IR in the same patient dataset showing enrichment in ribosome pathways. **d** RT-qPCR performed using primers specific to 851–961 ETS region show increased steady-state levels of rRNA, indicating increased Pol I activity after 4 Gy irradiation (IR-pink) compared to control (NIR-blue) in SUM1315. **e** Increased FUrd incorporation after 4 Gy irradiation (IR) corresponding to increase nascent rRNA synthesis in SUM1315. FUrd (green), Fibrillarin (red). The graph indicates fold change in mean fluorescence intensity, 100 cells quantified in each group. **f** Nucleoli were visualized using AgNOR staining and quantified as number of nucleoli per nucleus in SUM1315 cells before or after IR (4 Gy). Graph represents the % of cells with 1 or 2+ nucleoli/nucleus per field (**g**) RT-qPCR using primers specific to 851–961 ETS region shows increased steady-state levels of rRNA, indicating increased Pol I activity as early as 15 min to 4 h post IR (4 Gy) in SUM1315 cells. **h** Similar analysis of the ETS levels in HMEC following IR fails to show any specific increase. **i** Density gradient fractionation of SUM1315 4 h post irradiation (IR-pink) compared to control (NIR-blue). Polysome tracing was done by recording voltage over time with 40S, 60S, 80S, and polysome peaks denoted. Area under the curve was calculated for 80S (monosome) and polysome peaks for each sample set and a monosome-to-polysome ratio was calculated. **j** Colony-formation assay of SUM1315 irradiated with 1 Gy and seeded at 500 cells per well in triplicate. 10 nM BMH-21 was administered day 1 post seeding and media changed twice weekly during the course of the assay. Foci were stained and quantified using ImageJ software. Representative images of one well are depicted. Bar graphs indicate mean, the error bars are the standard error of the mean, and Student’s *t* test was used for statistical comparison with p values denoted (*n* = 3).

Radiation therapy comprises part of a multipronged approach to the standard of care for early, locally advanced, and/or metastatic breast cancer. Irradiation invokes systemic changes in ribosome biology, including potential alterations in rRNA transcription^11^. To further characterize the global impacts of radiation on rRNA biosynthesis, we utilized a publicly available dataset, GSE65505, which incorporates tumor profiles from a cohort of breast cancer patients pre- and post radiation. Gene set enrichment analysis (GSEA) of gene sets related to rRNA biosynthesis revealed a significant enrichment of genes important in various steps of rRNA transcription and processing (Fig. 1a and Supplementary Fig. 1A). Furthermore, GSEA revealed significant enrichment of gene signatures related to ribosome biology and RNA Pol I in patients post radiation therapy (Fig. 1b). We also identified enrichment of RNA Pol I regulatory gene sets in patient tumors post radiation (Supplementary Fig. 1B). KEGG and Reactome gene set databases also revealed significant enrichment of ribosome associated genes and genes involved in translation initiation, suggesting a systemic enhancement of rRNA biosynthesis and overall increase in ribosome biogenesis (Fig. 1c and Supplementary Fig. 1C). Informed by these correlations between rRNA biosynthesis genes and radiation therapy, we assessed the specific impact of radiation on RNA Pol I activation utilizing qPCR to measure the short-lived rRNA 5’-ETS transcripts (Supplementary Fig. 2A). Indeed, RNA Pol I activity increased twofold after irradiation in two independent breast cancer cell line models (Fig. 1d and Supplementary Fig. 2B). Complementary to this increase in 5’-ETS transcripts, nascent rRNA synthesis, as measured by FUrd incorporation, was also significantly increased post radiation (Fig. 1e and Supplementary Fig. 2C, D).

The nucleolus is a subnuclear non-membranous entity wherein rRNA transcription and processing occurs; as such, the nucleolar abundance reflects potential changes in rRNA biosynthesis. Irradiation increased the proportion of breast cancer cells with ≥2 nucleoli concomitant with the increase in rRNA transcription (Fig. 1f and Supplementary Fig. 2E). Next, we undertook detailed analyses to study the kinetics of RNA Pol I activity post radiation. This analysis revealed that activation of RNA Pol I occurs as early as 15 min post irradiation in both SUM1315 and SUM149 cells. While this is maintained at elevated levels through 4 h in SUM1315 cells, the RNA Pol I activity returns to baseline levels 1-hour post-irradiation in SUM149 cells (Fig. 1g and Supplementary Fig. 2F). This is not surprising since these tumor cells are derived from distinct patients and bear unique inherent molecular makeup. Unlike this elevation in RNA Pol I activity that we observed in triple-negative breast cancer (TNBC) cells (SUM1315, SUM149), we did not register any noteworthy changes in cell lines representing HER2+ or Luminal subtypes (SKBr3, MDA-MB-453, T47D, and MCF7) following IR. Normal/non-tumorigenic immortalized breast epithelial cells (HMEC and MCF10A) also failed to show an elevated response of RNA Pol I (Fig. 1h and Supplementary Fig. 2G). These findings indicate that upregulated RNA Pol I post-irradiation may be specific to TNBC. Guided by RNA Pol I transcriptional activity we explored the impact of radiation on the production of ribosomal subunits in breast cancer cells. While the polysome accumulation remained comparable, we observed a substantial increase in the area under the curve (AUC) for each respective peak of the 80S monosome after irradiation (Fig. 1i). This is important since the 80S monosome fraction contains mRNAs encoding low-abundance regulatory proteins, which are often transiently expressed at minimal levels, and these quite possibly serve as important mediators in various cellular responses, such as stress responses^12,13^. The majority of these monosomes were also shown to be in a state of translation elongation; this observation falls in line with the fact that our GSEA analysis supports enhanced translation elongation in patients post IR (Supplementary Fig. 1C)^12,13^. Next, we surmised that in order to survive irradiation, the tumor cells depend upon RNA Pol I activity. To test this, we evaluated cell survival with a colony-formation assay after irradiation in the presence or absence of the RNA Pol I inhibitor, BMH-21. As anticipated, cell survival was reduced after irradiation or RNA Pol I inhibition, but there was a significant reduction in colony formation of irradiated cells treated with BMH-21 after irradiation (Fig. 1j and Supplementary Fig. 2H). Collectively, these results demonstrate that survival of breast cancer cells is reliant on increased RNA Pol I transcriptional activity post radiation treatment.

### Irradiated breast tumor cells inhibited for RNA Pol I activity show a significantly decreased ability to establish pulmonary colonization

**Fig. 2:**
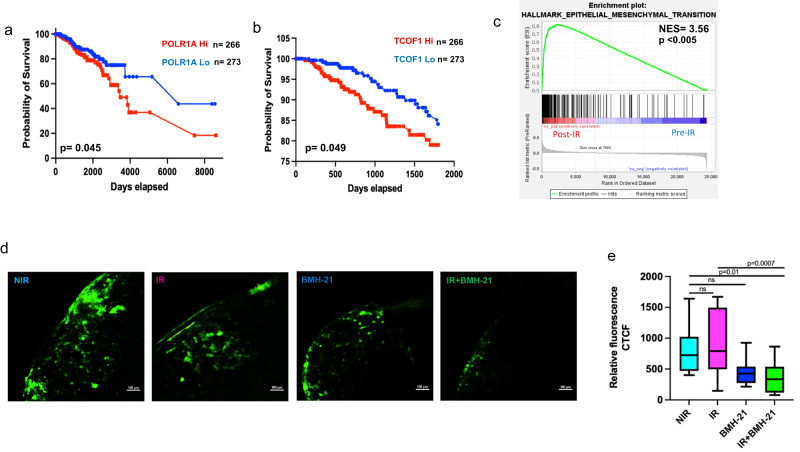
Irradiated breast tumor cells inhibited for RNA Pol I activity show a significantly decreased ability to establish pulmonary colonization. **a** TCGA breast cancer data-based KM survival curve for POLR1A expression. Graph shows significantly lower overall survival (OS) (*P* = 0.045) in patients expressing high levels of POLR1A. “*n*” denotes the number of patients in each group (**b**) TCGA breast cancer data-based KM survival curve for TCOF1 expression. Graph shows significantly lower overall survival (OS) (*P* = 0.049) in patients expressing high levels of TCOF1. **c** GSEA of Hallmark gene set epithelial-mesenchymal transition examined within tumors from 27 breast cancer patients before IR or 10 days after IR, graph shows enrichment of EMT signature in tumors post IR (NES = 3.56). **d** Ex vivo PuMA of irradiated 4T1 GFP-labeled cells. Following tumor cell injection, the lung sections were irradiated at 4 Gy and subsequently treated with 50 nM BMH-21. Six total sections were assessed per group with the image representative of a section. **e** Box plots with median (center line) and maximum and minimum values (whiskers) depict relative total corrected fluorescence analyzed from six sections per group. Student’s *t* test was used for statistical comparison with *P* values denoted (*n* = 6).

Ribosome biogenesis, specifically rRNA biosynthesis, is paramount for cell survival and unrestricted cellular proliferation. Cancer cells are metabolically very active and rely on increased demand for protein translation. Therefore, rRNA transcriptional activity is indispensable. RNA Polymerase I Subunit A (POLR1A) is the largest subunit of RNA Pol I and forms the catalytic core component of RNA Pol I^14^. Analysis of TCGA breast cancer patient data confirms a reliance of breast tumors on RNA Pol I activity as determined by a significant correlation of high POLR1A expressing tumors with decreased overall survival (Fig. 2a). In addition to its seemingly pivotal role in ribosomal DNA damage response, TCOF1 serves to regulate rRNA transcription through its interaction with UBF1. Similar to POLR1A, TCGA data revealed that elevated expression of TCOF1 predicts poor overall survival (Fig. 2b). More recent studies have continued to explore the importance of ribosome biogenesis as key driver of the metastatic cascade and not simply a byproduct^1^. The EMT program contributes to a number of fundamental steps in cancer progression, from invasion and migration to drug resistance^15^. We postulated that elevated rRNA biosynthesis, resultant from radiation therapy, may correlate with the EMT program. GSEA of tumors from breast cancer patients pre- and post radiation therapy displayed striking enrichment of the EMT gene set in patients after radiation therapy (Fig. 2c). Following this lead, we used pulmonary metastasis assay (PuMA) to assess the ability of tumor cells to colonize the lung, a common site of metastasis of breast cancer. The PuMA model recapitulates the initial stages of lung colonization of arrest at the secondary site, extravasation and survival at the secondary site, and formation of micrometastases, in the breast cancer metastatic cascade. This lung explant assay enabled us to evaluate the progression of tumor cells, in real time, to the formation of multicellular clusters, allowing us to recapitulate the complex cellular microenvironment of the metastatic site while preserving the three-dimensional structural integrity. Radiation alone did not appreciably impact the tumor cells’ ability to colonize the lung (Fig. 2d, e and Supplementary Fig. 3). Likewise, treatment with the RNA Pol I inhibitor, BMH-21 alone did not significantly impede lung colonization; however, BMH-21 treatment of irradiated lung tissue encumbered breast tumor cell colonization of lung tissue (Fig. 2d, e). As such, these data substantiate an important role for RNA Pol I activity in the ability of irradiated tumor cells to successfully establish outgrowth in the lungs.

### Irradiation induces activation and nucleolar localization of GLI1

**Fig. 3:**
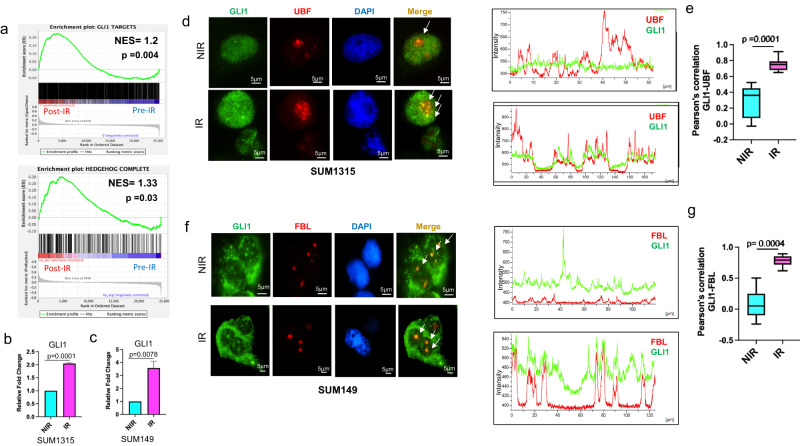
Irradiation induces activation and nuclear localization of GLI1. **a** GSEA of GSE65505 comparing pre- versus post-IR patient data sets showing enrichment of gene sets incorporating Chip-Seq validated GLI target genes “GLI1 targets” or a combined comprehensive list of Hedgehog Signaling gene set downloaded from https://www.gsea-msigdb.org/gsea/msigdb/genesets.jsp. RT-qPCR of **b** SUM1315 and **c** SUM149 cells post 4 Gy irradiation (IR-pink) compared to control (NIR-blue) demonstrating increased GLI1 transcripts. Bar graphs indicate mean, the error bars are the standard error of the mean, and Student’s *t* test was used for statistical comparison with *P* values denoted (*n* = 3). **d** Immunofluorescence staining of SUM1315 for GLI1, UBF, and DAPI in either NIR or 1 h post 4 Gy IR. Graphs represent fluorescence intensity profile for UBF and GLI1 showing overlapping patterns after IR but not in NIR. **e** Box plots with median (center line) and maximum and minimum values (whiskers) depict Pearson correlation of UBF and GLI1 colocalization is significantly higher in IR compared to NIR conditions. **f** Immunofluorescence staining of SUM149 for GLI1, FBL, and DAPI in either NIR or 1 h post 4 Gy IR. Graphs represent fluorescence intensity profile for FBL and GLI1 showing overlapping patterns after IR but not in NIR. **g** Box plots with median (center line) and maximum and minimum values (whiskers) depict Pearson correlation of FBL and GLI1 colocalization is significantly greater in IR compared to NIR conditions. Student’s *t* test was used for statistical comparison with *P* values denoted.

In irradiated cells, the GLI1 transcription factor of the Hh pathway is enriched at damaged rDNA loci and likely engages the NHEJ pathway to repair rDNA^9^. We hypothesized that GLI1 may play a fundamental role in activating RNA Pol I in response to irradiation. To explore the intersection of Hh activity and RNA Pol I activation, we analyzed the same cohort of patient data that demonstrated the enrichment of rRNA biosynthesis pathways. GSEA presented with an enrichment of both, GLI1 target genes and Hh pathway genes, in patient tumors post radiation therapy (Fig. 3a). This is further substantiated by a significant increase in the steady-state transcript levels of GLI1 in irradiated tumor cells (Fig. 3b, c). Concurrently, irradiated breast cancer cells display localization of GLI1 to the nucleolus as represented by clear colocalization of GLI1 and nucleolar markers UBF (UBTF) and Fibrillarin (FBL) (Fig. 3d, f). Furthermore, there is a distinct localization of GLI1 to nucleolar caps as evidenced by prominent colocalization of GLI1 with UBF1 post IR (Fig. 3d). Pearson’s correlation of GLI1 staining with UBF or FBL confirmed a significant overlap of signal intensities (Fig. 3e, g). To rule out a systemic effect of radiation on GLI nucleolar localization, we analyzed a non-tumorigenic cell line model, MCF10A. IR of MCF10A did not yield discernable localization of GLI1 to the nucleolar region, despite an overall increase in GLI1 staining, suggesting that GLI1 has a limited role in nucleolar DNA damage stress response in immortalized non-cancerous cells (Supplementary Fig. 4).

**Table 1:** Table of top hits identified from ChIP-MS.

UniProtKB name	UniProt ID	Entrez ID	ID	Quantitative value (normalized total spectra)						Fold change 2 h IR	Fold change 4 h IR
				NIR Isotype	NIR RPA194	2 h Isotype	2 h RPA194	4 h Isotype	4 h RPA194		
DNA-directed RNA polymerase I subunit	O95602	25885	RPA1	0	4.1037	0	4.4199	0	5.309	1.077052416	1.293710554
Zinc finger protein	P08151	2735	GLI1	0	6.1556	0	9.5764	0	11.208	1.555721619	1.820781077
Nucleolar transcription factor 1	P17480	7343	UBTF	0	0.75261	0	0	0	1.5405	0	2.046876868
Nibrin	O60934	4683	NBN	0	0	0	1.4733	0	1.7697	12	12
Treacle protein	Q13428	6949	TCOF1	0	3.0778	0	6.6298	0	5.8989	2.15407109	1.91659627
N-acetyltransferase 10	Q9H0A0	55226	NAT10	0	1.5389	0	2.2099	0	2.3595	1.436025733	1.533238027
HEAT repeat-containing protein 1	Q9H583	55127	HEATR1	0	1.0259	0	0.73664	0	3.5393	0.718042694	3.449946389
DNA repair protein RAD50	Q92878	10111	RAD50	0	4.1037	0	1.4733	0	6.4887	0.359017472	1.581182835
Histone acetyltransferase	Q92794	7994	KAT6A	0	0	0	1.1881	0	0	12	0

RPA194 was chromatin immunoprecipitated from SUM1315 cells 2 h and 4 h post 4 Gy irradiation followed by LCMs analysis to identify proteins bound at rDNA loci. Normalized total spectra were used to calculate fold change between IR and NIR groups, which contained no peptides in isotype controls.

### GLI1 facilitates recovery of RNA Pol I activity in irradiated tumor cells

**Fig. 4:**
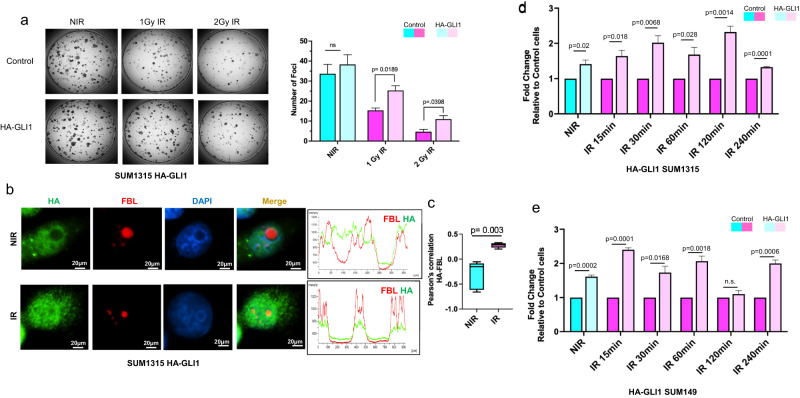
GLI1 facilitates recovery of RNA Pol I activity in irradiated tumor cells. **a** Colony-formation assay of SUM1315 HA-GLI1 or control cells irradiated with 1 Gy or 2 Gy and seeded at 500 cells per well in triplicate. Foci were stained and quantified using ImageJ software. Representative images of one well are depicted. **b** Immunofluorescence staining of SUM1315 HA-GLI1 cells for HA, FBL, and DAPI in either NIR or 1 h post IR (4 Gy) conditions. The graph represents fluorescence intensity profile for FBL and HA showing overlapping staining after IR but not in NIR conditions. **c** Box plots with median (center line) and maximum and minimum values (whiskers) depict the Pearson correlation of FBL and HA staining colocalization. Student’s *t* test was used for statistical comparison with *P* values denoted. **d** RT-qPCR using primers specific to 851–961 ETS region shows elevated levels of steady-state rRNA at baseline (NIR) and at multiple intervals following IR indicative of increased Pol I activity in SUM1315 HA-GLI1 or **e** SUM149 HA-GLI1 cells. Bar graphs indicate mean, the error bars are the standard error of the mean, and Student’s *t* test was used for statistical comparison with *P* values denoted (*n* = 3).

Given that GLI1 localizes to the nucleolus in such a short time, we evaluated whether the cells need to produce GLI1 protein de novo. To test this, we blocked protein synthesis with cycloheximide. Cells pre-treated with cycloheximide demonstrate effective nucleolar localization only after IR (Supplementary Fig. 5A, B), indicating that the available pool of endogenous GLI1 is sufficient to make the transition to the nucleolus in response to IR, without a need for newly synthesized GLI1 protein. Inhibiting GLI activity compromised the ability of tumor cells to reactivate RNA Pol I^9^. We postulated that GLI1 would provide a survival advantage to irradiated tumor cells. So, we tested the effect of ectopically expressing GLI1 in SUM1315 cells. SUM1315 cells engineered to stably express HA-tagged GLI1 displayed a significant increase in colony formation after IR compared to non-irradiated control cells (Fig. 4a). Similar to endogenous GLI1, HA-tagged GLI1 also demonstrated nucleolar localization only upon IR (Fig. 4b, c).

Post IR there is a gradual increase in RNA pol I activity (Fig. 1). To assess the impact of GLI1 expression on RNA Pol I transcriptional activity after IR, we evaluated RNA Pol I activity at multiple time intervals following irradiation of control and GLI1-expressing cells. At baseline non-irradiated tumor cells engineered to ectopically express GLI1 demonstrate a 1.5-fold increase in RNA Pol I activity. In response to IR, these cells show a robust increase in RNA Pol I activity (Fig. 4d, e), thus demonstrating that GLI1 facilitates activation of rRNA synthesis after IR.

### TCOF1 facilitates nucleolar translocation of GLI1 in irradiated cells

**Fig. 5:**
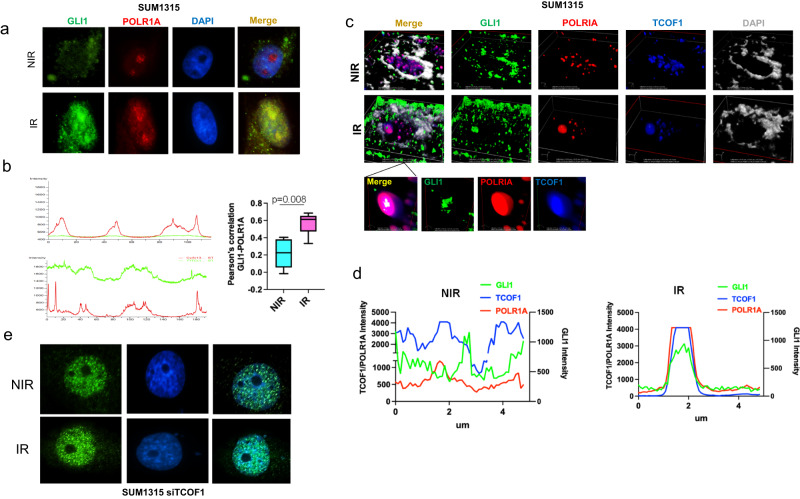
TCOF1 facilitates nucleolar translocation of GLI1 in irradiated cells. **a** Immunofluorescence staining of SUM1315 cells for GLI1 (green), POLR1A (red), and DAPI (blue) in either NIR or 4 h post 4 Gy IR conditions. **b** Adjacent graph shows the fluorescence intensity profile for specific staining across a linear path through the image. Box plots with median (center line) and maximum and minimum values (whiskers) depict Pearson correlation for staining colocalization of POLR1A and GLI1 is significantly higher IR compared to NIR conditions. Student’s *t* test was used for statistical comparison with p values denoted. **c** 3D confocal images of NIR (top) and IR (bottom) SUM1315 cells labeled with GLI1 (green), RPA194 (red), TCOF1 (blue), and DAPI (gray) depict increased nucleolar localization and overlapping localization of GLI1, RPA194, and TCOF1 4 h post IR in the nucleolar cap compartment. **d** Graphs represent the florescence intensity profile for RPA194, TCOF1 and GLI1 showing overlapping patterns in nucleolar compartment after IR but not in NIR. **e** Immunofluorescence of GLI1 (green) in SUM1315 cells silenced for TCOF1 in both, control and irradiated cells (4 h post IR).

To gain further insight into the role of GLI1 in regulating RNA Pol I transcription activation, we sought to determine if GLI1 might co-localize with the major RNA Pol I subunit, POLR1A. Only upon IR, could we detect GLI1 in the nucleolar space that is co-occupied by POLR1A (Fig. 5a, b and Supplementary Fig. 6A). To further characterize the molecular underpinnings of a potential role for GLI1 in facilitating re-activation of RNA Pol I, we utilized an approach combining chromatin immunoprecipitation followed by LC–MS (ChIP-MS), wherein, irradiated tumor cells were subjected to ChIP using POLR1A followed by mass spectrometric analysis of proteins that co-immunoprecipitated with POLR1A at the rDNA loci (Supplementary Fig. 6B). Isotype controls were used to normalize for background signal and only those proteins differentially identified in POLR1A ChIP were compared between control (non-irradiated) and irradiated groups. As expected, POLR1A was the top hit in the proteomics analysis but demonstrated no appreciable change in abundance upon irradiation. We also registered an increase in the enrichment of the main transcription factor for RNA Pol I, Upstream Binding Transcription Factor (UBTF1). Expectedly the number of DNA damage response proteins were significantly increased 2 h and 4 h after radiation. Notably, following IR, GLI1 was enriched with POLR1A further solidifying our evidence thus far. Moreover, we found that TCOF1 was enriched after radiation (Table 1). Previous studies have identified a prominent role of TCOF1 in the rDNA damage response, concluding that TCOF1 acts to recruit Nibrin (NBS1) to sites rDNA damage^16^. Building upon this evidence, we assessed the localization of GLI1 and TCOF1. In irradiated cells, GLI1 and TCOF1 demonstrated colocalization by immunofluorescence, suggesting that TCOF1 may play a role in trafficking GLI1 to the nucleolus (Supplementary Fig. 6C, D). To gain a better understanding and to expand upon this observation, we irradiated tumor cells and stained for GLI1, POLR1A, and TCOF1. Confocal imaging clearly identified a striking overlap of all three proteins in the nucleolus after radiation (Fig. 5c and Supplementary Fig. 6E) supported by intensity signal plots that reveal a stark concurrence of all three signal intensities in irradiated cells (Fig. 5d and Supplementary Fig. 6F). To definitively determine the role of TCOF1 in mediating GLI1 localization to the nucleolus after radiation, we silenced TCOF1 using small interfering RNA (Supplementary Fig. 6G). This severely compromised the nucleolar localization of GLI1, providing evidence for TCOF1 mediating the translocation of GLI1 to the nucleolus in irradiated cells (Fig. 5e and Supplementary Fig. 6H).

### GLI1 overrides the inhibitory effects of DNAPKc and ATM inhibition to sustain RNA Pol I activation

Among the proteins that were co-immunoprecipitated with POLR1A and GLI1 were the DNA damage repair proteins NBS1 and RAD50 Double Strand Break Repair Protein (RAD50 (Table 1), both of which are components of the heterotrimeric MRE11-RAD50-NBS1 (MRN) complex, which assumes a central role in repairing double-strand breaks in DNA. In response to rDNA double-strand breaks, NBS1 is recruited to nucleolar foci by TCOF1 and functions as a member of the MRN complex and navigates damaged rDNA foci to nucleolar caps^17^. In the process of nucleolar localization of NBS1, the ATM serine/threonine kinase (ATM) plays an important role by phosphorylating and enabling TCOF1 to transport NBS1^17^. The DNA double-strand break response through NHEJ and HR is mainly mediated by DNA-PKcs, the catalytic subunit of the DNA-dependent protein kinase (DNA-PK), and ATM respectively. Together with the KU proteins, DNA-PKcs form the DNA-PK holoenzyme which is a classical NHEJ factor.

**Fig. 6:**
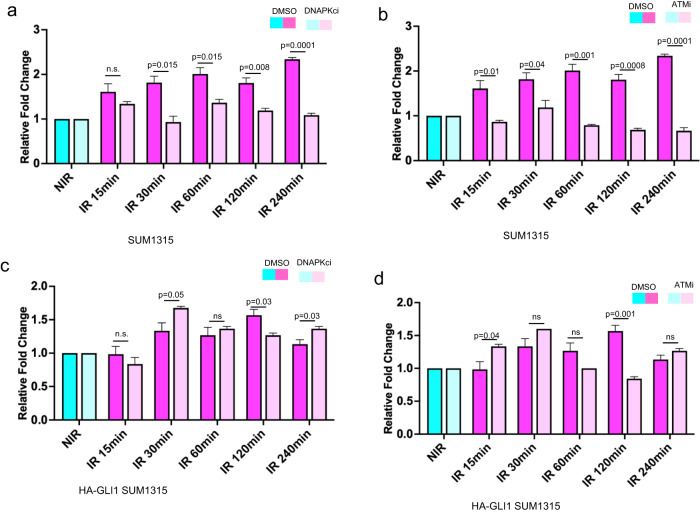
GLI1 overrides the inhibitory effects of DNAPKc and ATM inhibition to sustain RNA Pol I activation. **a** RT-qPCR of the 851–961 5’ ETS region shows elevated 5’ETS rRNA transcripts, indicating increased RNA Pol I activity after irradiation (IR-pink) compared to control (NIR-blue). Treatment with 2.5 µM DNAPKc i (light pink) decreases RNA Pol I activity after IR compared to control (dark pink) in SUM1315. **b** RT-qPCR of 851–961 5’ ETS region shows increased steady-state levels of rRNA, indicative of elevated RNA Pol I activity after 4 Gy irradiation (IR-pink) compared to control (NIR-blue). Treatment with 5 µM ATM i (light pink) blocks RNA Pol I activation after IR compared to control (dark pink) in SUM1315. **c** RT-qPCR of 851–961 5’ ETS region in HA-GLI1 SUM1315 indicating increased RNA Pol I activity after irradiation (IR-pink) compared to control (NIR-blue). This increase in RNA Pol I activation after IR compared to control (dark pink) persists in the presence of 2.5 µM DNAPKc i (light pink) in SUM1315 HA-GLI1 (**d**) RT-qPCR of 851–961 5’ ETS region in HA-GLI1 SUM1315 indicating increased RNA Pol I activity after 4 Gy irradiation (IR-pink) compared to control (NIR-blue). Increased RNA Pol I activity after IR compared to control (dark pink) persists with 5 µM ATM i (light pink) in SUM1315 HA-GLI1. Bar graphs indicate mean, the error bars are the standard error of the mean, and Student’s *t* test was used for statistical comparison with *P* values denoted (*n* = 3).

In order to investigate if activation of RNA Pol I is contingent upon the nucleolar DNA damage stress response, we used small molecule inhibitors of ATM (KU-55933) and DNA-PK (NU7441) (Supplementary Fig. 7A). Inhibiting DNA-PK augmented radiation-induced DNA damage as evidenced by the rapid appearance of Phospho-Histone H2A.X (γH2AX) as early as 1 h after treatment with NU7441 (Supplementary Fig. 7B) and blunted recovery of RNA Pol I activity (Fig. 6a and Supplementary Fig. 7D). Likewise inhibiting ATM notably reduced the levels of phosphorylated (S824) Tripartite Motif Containing 28 (KAP-1) which is an ATM substrate^18^ (Supplementary Fig. 7C) and inhibited activation of RNA Pol I (Fig. 6b and Supplementary Fig. 7E). Cells engineered to ectopically express GLI1 overcame the inhibitory effects of ATM and DNA-PK inhibition and RNA Pol I activity remained elevated post radiation (Fig. 6c, d and Supplementary Fig. 7F, G). Next, we tested the effects of Vismodegib, a clinically approved Hh/GLI inhibitor^19^, and the DNA-PKcs inhibitor, M3814 on irradiated breast cancer cells. M3814 sensitizes cancer cell lines to ionizing radiation^20^ and is well-tolerated in a phase I clinical trial^21^. Vismodegib alone was ineffective in sensitizing tumor cells to radiotreatment, while M3814 demonstrated modest efficacy. In combination with Vismodegib, M3814 demonstrated synergistic activity (combination index <1.0) in irradiated tumor cells (Supplementary Fig. 7H). These results collectively provide evidence that GLI activity sanctions tumor cells to activate RNA Pol I even when nucleolar DNA damage response is interrupted, and inhibiting GLI activity remarkably sensitizes irradiated tumor cells to DNA-PK inhibition.

### GLI1 regulates histone acetylation at the RNA Pol I promoter to facilitate activation after irradiation

**Fig. 7:**
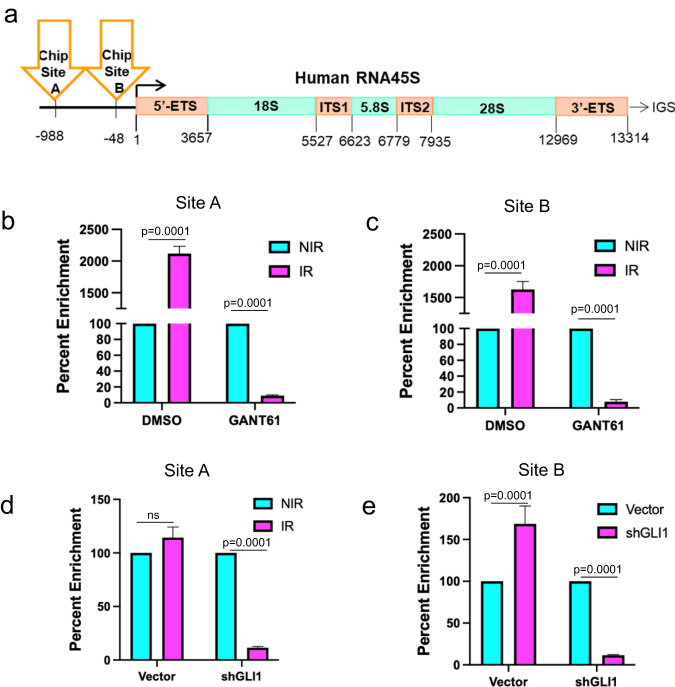
GLI1 regulates histone acetylation at the RNA Pol I promoter to facilitate recovery of RNA Pol I activity in irradiated tumor cells. **a** Schematic of Human 45S rDNA repeating unit: ETS external transcribed spacer, ITS internal transcribed spacer, IGS intergenic sequence. Primers used for to amplify putative histone acetylation sites in the promoter element of the 45S rDNA are indicated. **b** H3K27ac ChIP RT-qPCR 4 hr post 4 Gy irradiation (pink) compared to control (blue) of rRNA at -988 or **c** -48 sites in SUM1315 DMSO or 10 µM GANT treated cells. **d** H3K27ac ChIP RT-qPCR 4 hr post 4 Gy irradiation (pink) compared to control (blue) of rRNA at -988 or **e** -48 sites in SUM1315 vector or shGLI1 cells. Bar graphs indicate mean, the error bars are the standard error of the mean, and Student’s *t* test was used for statistical comparison with *P* values denoted (*n* = 3).

Epigenetic modification of rDNA loci serves as a key regulatory node for rRNA transcription. Previous ChIP-seq studies have identified regions of the rDNA loci that regulate transcription through methylation and acetylation of histones^22,23^. Our ChIP-MS revealed that concurrent with GLI1 and POLR1A, N-acetyltransferase 10 (NAT10) and histone acetyltransferase (KAT6A) were enriched at rDNA loci upon irradiation (Table 1). Led by this evidence we assessed a chromatin mark demonstrated to indicate transcriptionally active rDNA loci^22^. We assessed two sites in the rDNA proximal promoter region for enrichment of H3K27 acetylation by ChIP followed by PCR of a site at −48 or −988 (Fig. 7a). H3K27ac was significantly enriched in irradiated tumor cells at both sites in the rDNA promoter (Fig. 7b, c). Inhibition of GLI1, either through GANT61 (Fig. 7b, c) or gene silencing (Fig. 7d, e), dramatically reduced H3K27 acetylation at both sites in the rDNA promoter region. Reduction of these key histone acetylation sites on the rDNA loci in the absence of GLI activity, provides further insight into the mechanism by which GLI activates RNA Pol I following radiation.

### RNA Pol I and Hh activity are important for pulmonary colonization of irradiated breast cancer cells

**Fig. 8:**
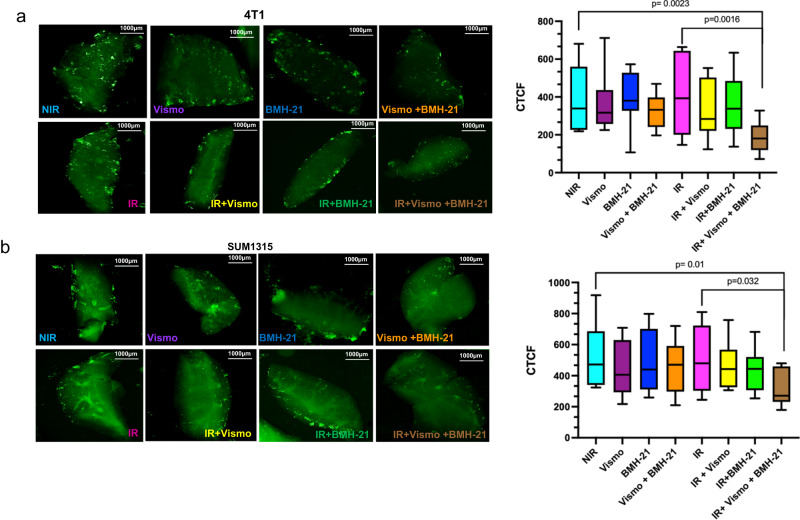
RNA Pol I and Hh activity are important for pulmonary colonization of irradiated breast cancer cells. **a** Ex vivo PUMA of 4T1 GFP-labeled cells or **b** SUM1315 GFP-labeled cells. Following tumor cell irradiation at 4 Gy, cells were injected via tail vein, lungs harvested, and subsequently treated with 50 nM BMH-21, 5 µM Vismodegib alone, or a combination of both, with and without IR. Six total sections were assessed per group. The image represents a single lung section from each group. Box plots with median (center line) and maximum and minimum values (whiskers) depict relative total corrected fluorescence analyzed from six sections per group. Student’s *t* test was used for statistical comparison with *P* values denoted (*n* = 6).

In order to test the relevance of GLI on the recovery of irradiated tumor cells, we treated breast cancer cells with Vismodegib^19^, in a short-term cell viability assay. BMH-21 or Vismodegib alone did not significantly affect the overall viability of irradiated tumor cells. However, in combination, BMH-21 and Vismodegib demonstrated a significant reduction in the viability of irradiated tumor cells with a combination index that indicates synergy (Supplementary Fig. 8A). As such, inhibiting Hh activity and RNA Pol I activity augment the deleterious effects of radiation on tumor cells. Next, we adopted the PuMA to evaluate the formation of multicellular clusters of tumor cells in the lung. Neither BMH-21 nor Vismodegib, taken alone or in combination impacted tumor cell outgrowth of non-irradiated SUM1315 cells. While Vismodegib-treatment of irradiated cells did not seem to have a significant impact, BMH-21 caused a notable decline in the colonized clusters (Fig. 8a). The combination of Vismodegib and BMH-21 had the best outcomes with a significant decrease in colonies of irradiated cells compared to the irradiated cells alone (Fig. 8b and Supplementary Fig. 8B). Taken together, the data provide evidence that inhibiting RNA Pol I and Hh activity mitigate the ability of irradiated tumor cells to recover from the stress of IR presenting a new opportunity to enhance the effectiveness of radiation treatment (Fig. 8c).

### Inhibiting ribosome biosynthesis and Hh activity enhances the effectiveness of radiotherapy

**Fig. 9:**
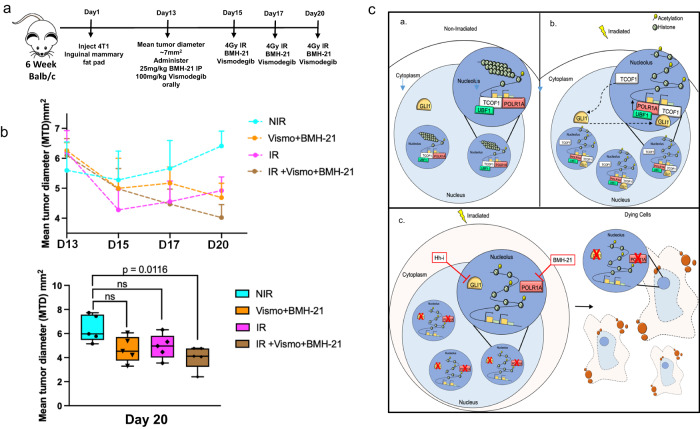
Ribosome biosynthesis and Hedgehog activity cooperatively present as actionable signaling mechanisms in breast cancer following radiotherapy. **a** Schematic of the experimental timeline. **b** Established tumors (day 13) were irradiated with 12 Gy total over three treatments, in addition to treatment with BMH-21 (25 mg/kg, IP) and Vismodegib (2 mg, oral). Tumor growth was measured by calipers Line graphs indicate mean and the error bars are the standard error of the mean. Inset box plot with median (center line) and maximum and minimum values (whiskers) depicts tumor diameter measurement at day 20. Tukey’s multiple comparisons was used for statistical comparison with *P* values denoted (*n* = 5). **c** Schematic representation of a breast cancer cell in (**a**) non-irradiated state, (**b**) irradiated state, and (**c**) irradiated in the presence of hedgehog inhibitor (Hh-i) and RNA Pol I inhibitor (BMH-21). **a** When the cells are not exposed to IR, GLI1 is in the nucleus (light blue) and absent in the nucleoli (dark blue). The ribosomal DNA is transcribed by POLR1A with TCOF1 and UBF1. **b** Upon cells exposure to IR, GLI1 enters the nucleolus with TCOF1 and works with POLR1A, TCOF1 and UBF1 to enhance Pol I transcription activity and facilitate cell survival. **c** Inhibiting GLI1 and RNA Pol I disables the survival machinery and sensitizes the tumor cells to IR.

Given that irradiated tumor cells treated with a combination of Vismodegib and BMH-21 struggled to efficiently colonize the lung, we assessed this treatment approach in vivo. We utilized a syngeneic 4T1 murine model, and initiated treatment after tumors were ~7 mm in diameter (Fig. 9a), after which mice tumors were irradiated for 3 days (4 Gy) for a total of 12 Gy in addition to treatment with Vismodegib and BMH-21. Vismodegib combined with BMH-21 had a modest effect on tumor growth, which was similar to that of irradiated tumors alone, albeit neither significant (Fig. 9b). Conversely, irradiated tumor progression was significantly blunted when combined with treatment of BMH-21 and Vismodegib (Fig. 9b). Altogether, these data establish that Hh/GLI activity and RNA Pol I activity are essential for tumor cells to resurge from radiotherapy, and consequently, present as new actionable vulnerabilities in breast tumor cells to enhance the effectiveness of radiation treatment (Fig. 9c).

## Discussion

rDNA is the most transcribed region in the human genome. Due to the hundreds of rDNA repeating units, this region is acutely prone to aberrations and is extremely vulnerable to therapeutic insults that target DNA^24^. Loss of rDNA integrity leads to repression of RNA Pol I activity. Re-activation of RNA Pol I is an obligatory step for cells to invigorate and survive this stress. This is underscored in our unbiased analysis from patients who received radiation treatment for breast cancer. Tumors that were obtained post-irradiation also showed concomitant enrichment of a gene signature of EMT. Our studies have demonstrated that inhibiting RNA Pol I with the small molecule inhibitor BMH-21, significantly reduced the ability of tumor cells to survive radiation-induced death and mitigated metastatic colonization in a pulmonary metastasis explant assay, further solidifying our proposition that the activity of RNA Pol I is indispensable for tumor cells to recover from radiation-induced cell death. Led by this evidence, we identified a novel mechanism that facilitates the recovery of RNA Pol I activity in irradiated cells. We present that activation of RNA Pol I following irradiation is associated with TCOF1-mediated recruitment of the Hh transcription factor, GLI1, into the nucleoli. Transport of GLI1 into the nucleolus occurs rapidly and is not dependent on de novo synthesis of GLI1. In the nucleolus GLI1 associates with POLR1A, the ribosomal subunit of RNA Pol I, and maintains the nucleolar chromatin in an open state to facilitate RNA Pol I activity.

In response to DNA double-strand breaks, there is silencing of rRNA transcription associated with rapid and transient recruitment of NBS1 into the nucleoli through a process that invokes TCOF1^16^. Aligned with this, we registered NBS1 in the nucleolus associated with POLR1A only upon irradiation. This complex also includes RAD50, albeit at a lower abundance, cumulatively indicating an enrichment of repair proteins in the irradiated nucleoli. NBS1 is a core component of the conserved MRN complex. The MRN complex controls biological outcomes of DNA damage by acting as flexible scaffold, combined sensor, and a signaling and effector complex. Importantly, the MRN complex navigates the choice of DNA repair by interacting with DNA-PK that engages NHEJ repair. Also, by recruiting ATM, the MRN complex promotes chromatin remodeling and exerts control over the cell cycle^25^.

As anticipated, inhibiting ATM or DNA-PK significantly impeded RNA Pol I activity, but this impedance was overcome when the cells were engineered to ectopically express GLI1, suggesting that GLI1 may impact RNA Pol I directly and/or indirectly through overriding the effects of a blunted nucleolar DNA damage response. Hh/GLI activity has been recognized to increase the tolerance for damaged DNA by attenuating the DNA damage response^9,26,27^. Our findings identify that in the irradiated nucleolus, GLI1 is a member of a multi-protein complex that associates with POLR1A. This complex also includes chromatin modifiers, KAT6A and NAT10. KAT6A is a histone acetyltransferase that can catalyze H3K9Ac, H3K14Ac, and H3K23Ac, with a well-characterized role in acute myeloid leukemia^28,29^. NAT10 is a member of the Gcn5-related family of histone acetyltransferases and is located in the nucleolus. By acetylating histone and UBF, NAT10 activates RNA Pol I transcription^30^ and also regulates telomerase biogenesis and length^31^. Inhibiting Hh/GLI activity significantly decreased histone acetylation in the regulatory region of RNA Pol I providing further evidence that in the nucleolus, GLI1 facilitates chromatin remodeling to enhance RNA Pol I activity following irradiation.

Despite the benefits of radiation therapy in controlling locoregional recurrence and metastasis, resistance remains a major challenge. Radioresistance is acquired by tumor cell-intrinsic and extrinsic (microenvironmental) alterations. From the context of intrinsic changes, increasing evidence has suggested the role of EMT in multiple tumor types. The EMT process promotes invasiveness, migration, and radioresistance^32^. Ribosome biogenesis enables a program of mesenchymal gene expression that enables cellular plasticity and invasion^33^. As such, it was impressive that our unbiased assessment of breast tumors post-radiotherapy showed enrichment of signatures of ribosome biosynthesis and hallmarks of EMT concurrent with a Hh program, presenting these mechanisms as cytoprotective pathways that enable tumor cells to emerge as a radioresistant population that typically has a high potential to recur and/or metastasize.

Breast cancer has a propensity to locoregionally recur in the chest wall and/or metastasize to the lungs, bone, and/or liver. In particular, up to 40% of patients with TNBC can present with metastasis to the lungs^34^. The Hh inhibitor Vismodegib is used in the clinic for treatment of basal cell carcinoma and is being evaluated in clinical trials for head and neck cancer, prostate cancer, multiple myeloma, and other cancers. In locally advanced basal cell carcinoma the addition of radiotherapy to Vismodegib induction therapy offered prolonged progression-free survival^35^. Our investigations present that Vismodegib and BMH-21 target the dependence of irradiated breast cancer cells and disable the metastatic outgrowth of breast cancer cells in the lungs. As such, our investigations revealed that following radiotherapy, ribosome biosynthesis and Hh activity present as actionable signaling mechanisms, and inhibiting RNA Pol I and Hh activity in patients receiving radiation therapy may provide a significant treatment advantage over current treatment modalities for breast cancer.

## Methods

### Cell culture

SUM1315 cells (Asterand Bioscience, Detroit, MI) were maintained in DMEM/F-12 (Thermo Fisher, Waltham, MA) containing 5% FBS (Thermo Fisher), 10 µg/ml insulin (Sigma Aldrich, St. Louis, MO), and 25 ng/ml epidermal growth factor (Millipore Sigma, St. Louis MO). SUM149 (Asterand Bioscience, Detroit, MI) cells were maintained in DMEM/F-12 (Thermo Fisher) containing 5% FBS (Thermo Fisher), 10 µg/ml insulin (Millipore Sigma), and 1 µg/ml hydrocortisone (Millipore Sigma).

T47D (ATCC #HTB-133) cells were cultured in RPMI-1640 media (Thermo Fisher) supplemented with 10%FBS (Thermo Fisher) and 10 μg/ml insulin (Millipore Sigma, St. Louis, MO). MCF10A (generously gifted by Dr. Fred Miller, Karmanos Cancer Institute) were cultured in DMEM/F-12 supplemented with 5% Horse Serum (Thermo Fisher), 10 μg/ml insulin (Millipore Sigma), 25 ng/ml hEGF (Millipore Sigma), 250 ng/ml hydrocortisone (Millipore Sigma), and 100 ng/ml cholera toxin (Millipore Sigma). SK-BR-3 (ATCC #HTB-30) cells were grown in McCoy’s 5 A (Thermo Fisher) supplemented with 10% FBS. HMECs cells (generously gifted by Dr. Robert Weinberg) were cultured in serum-free DMEM/F-12 (Thermo Fisher) media supplemented with 10 ng/ml hEGF, 500 ng/ml hydrocortisone and 10 µg/ml insulin.

Stable GLI1-expressing SUM1315 or SUM149 cells were generated by transfecting pcDNA3.1 HA-GLI1 or an empty-vector (pcDNA3.1) using Lipofectamine 2000 (Thermo Fisher) according to the manufacturer’s protocol and selected with G418 (Thermo Fisher) (200 µg/mL). Culture media were free of antibiotics and antimycotics unless otherwise stated. Cells were maintained at 37 °C in a humidified environment containing 5% CO_2_.

### In vivo experiments

Luciferase-expressing 4T1 cells (2.5 × 10^5^) were suspended in Hanks' Balanced Salt Solution and injected into the inguinal mammary fat pad of 8-week-old female Balb/c mice. Tumor growth was documented by caliper measurements and mean tumor diameter was calculated. Once tumors attained a diameter of ~7 mm, mice were administered 100 μL (2 mg/mouse) of Vismodegib (100 mg/kg) or DMSO as a vehicle control by oral gavage and BMH-21 (25 mg/kg) or vehicle control through intraperitoneal injection thrice weekly for 2 weeks with irradiation delivered to the mammary tumor using 3 doses of 4 Gy each on alternate days, for a total of 12 Gy. Mice euthanasia (carbon dioxide inhalation confirmed by cervical dislocation) is carried out per the guidelines of the American Veterinary Medical Association (AVMA), when a mean tumor diameter of 100 mm^2^ is attained. For euthanasia, prior to cervical dislocation, mice are placed in a chamber, then 100% CO_2_ is introduced at a fill rate of 30–70% of the chamber volume per minute (for at least 3 min) to induce rapid unconsciousness with minimal distress to the animals. All animal studies were approved and conducted in accordance with the University of Alabama at Birmingham Institutional Animal Care and Use Committee.

### Irradiation

Mice were irradiated using a small animal irradiator (Precision X-ray X-RAD 320) with a 320 kVp Orthovoltage Energy X-ray unit. Exposures were quantified using the UNIDOS E dosimeter (PTW). Dose output for this x-ray system: 1.28 Gy/min at 320 KV, 12.5 mA, 50 cm SSD, (HVL ≈ 1 mm Cu). Mice were anesthetized by intraperitoneal injection of ketamine (100 mg/kg) and xylazine (10 mg/kg), then placed under a 2-cm-thick Cerrobend block with four wedge-shaped openings allowing irradiation only to the left mammary fat pad tumor region (total time to deliver 4 Gy dose = 188 s). Where applicable, tumor cells were irradiated with the indicated doses using the X-RAD 320 X-ray irradiator.

### Plasmids

GLI1 was amplified from cDNA to generate HA-tagged GLI1 utilizing the following primers: forward: ATA AGA ATG CGG CCG CCATGTACC CATACG ATG TTC CAG ATT ACG CTC TTT TCA ACTCGA TGA CCC CA; reverse: TGC TCT AGA TTA GGC ACT AGA GTT GAG. The HA-GLI1 fragment was subsequently cloned into *NotI* and *XbaI* sites of pcDNA3.1+ (Thermo Fisher).

### Real-time PCR

Cells were exposed to 4 Gy irradiation or treated with 2.5 µM NU7441 (DNA-PKcs i) or 5 µM KU-55933 (ATM i) 1 h prior to irradiation, followed by RNA extraction at times indicated post-irradiation, using the RNeasy Mini Kit (Qiagen, Hilden, Germany). All non-irradiated controls were collected in conjunction with the 1 h post-irradiated cells. cDNA was generated using 1 µg total RNA and the High Capacity cDNA kit (Thermo Fisher). Real-time PCR was performed using 40 ng total cDNA per reaction, 2× TaqMan Fast Advance Master Mix or Maxima 2X SYBR Green Master Mix (Thermo Fisher), and the following primers: TCOF1 For- CGG GAG CTA CTT CCC CTG AT; Rev- CAG AAG GGT TAC GGG CTG AG and ACTB For- CATGTACGTTGCTATCCAGGC; Rev-CTCCTTAATGTCACGCACGAT or TaqMan primer probes: β-actin or GLI1 (Thermo Fisher).

The rate of RNA Pol I transcription was measured by determining short-lived 5’ external transcribed spacer (5’ETS) rRNA of the 47S pre-RNA by real-time PCR. Reactions were performed with 2 µl of 1:50 diluted cDNA with 2× Maxima SYBR Green Master Mix (Thermo Fisher) along with the following primer sets (11)(11)(7)- 5’ETS 851–961 forward: GAACGGTGGTGTGTCGTT; reverse: GCGTCTCGTCTCGTCTCACT.

Reactions were run in triplicate using Applied Biosystems StepOnePlus Real-time PCR machine. Analysis was done using ^ΔΔ^CT to determine relative fold changes in mRNA or 5’ETS transcripts.

### rRNA synthesis assay

rRNA synthesis was measured as a readout of FUrd incorporation into nascent rRNA transcripts. Briefly, cells were seeded onto glass coverslips following 4 Gy irradiation and allowed to attach overnight. Cells were pulsed with 2 mM FUrd (Millipore Sigma) for 10–20 min. Following the pulse, cells were immediately fixed with 3.7% formaldehyde for 10 min at room temperature. Coverslips were subsequently washed and permeabilized with 0.3% Triton X-100 for 15 min, blocked in 5% BSA, and incubated with anti-Brdu 1:400 (Millipore Sigma, B8434) and anti-Fibrillarin 1:400 (Abcam, Cambridge UK, ab166630) overnight at 4 °C. Coverslips were incubated with appropriate Alexa Fluor 488 or Alexa Fluor 594 secondary antibodies 1:400 (Thermo Fisher) and mounted using Vectashield Plus with DAPI (Vector Labs, Newark, California).

Images were acquired with a Nikon Eclipse Ti-U using the same exposure times for all images (Nikon Instruments Inc., Melville, NY). Mean Fluorescence Intensity was determined using NIS-Elements Advanced Research software analyzing 100 cells across 10 random fields. Representative images are depicted.

### Nucleolar quantification

Cells were plated on coverslips then exposed to 4 Gy irradiation, and fixed in 3.7% formaldehyde 4 h post IR. Bright-field images were acquired using Nikon Eclipse Ti-U and NIS elements software, and the number of nucleoli per individual nucleus were quantified and analyzed using GraphPad Prism version 8 (GraphPad Software, La Jolla, CA).

### Polysome profiling

SUM1315 cells were exposed to 4 Gy irradiation and 4 h post IR, cells were treated with 100 µg/ml cycloheximide (Millipore Sigma) for 5 min and washed three times with cold 1x PBS containing 100 µg/ml cycloheximide. Cell lysates were prepared by scraping cells into polysome lysis buffer (20 mM Tris pH 7.4, 10 mM MgCl_2_, 300 mM NaCl, 1% Triton X-100, 100 µg/ml cycloheximide, 0.1U/µl RNase inhibitor (Thermo Fisher), protease inhibitor cocktail (Thermo Fisher), 0.5 mg/ml heparin (Thermo Fisher), rotating 10 min at 4 °C, and spun at 13,000 RPM for 10 min. Equal protein was layered over 10% to 50% sucrose density gradients and sedimented using a Beckman SW41Ti rotor at 35,000 rpm for 3 h 20 min at 4 °C. Gradients were fractionated and collected (16 s, 350 µl/fraction), and the absorbance at 254 nm was recorded continuously using a Brandel BR-188 density gradient fractionation system. The area under the curve for 80S and polysome peaks was calculated using Peak Chart analysis software (Brandel) and depicted as a ratio of monosomes to polysomes.

### Colony-formation assay

SUM1315 and SUM149 cells were irradiated (1 Gy) and seeded in triplicate in six-well plates at a density of 500 or 100 cells per well, respectively. 24 h after seeding, media was gently aspirated and replaced with complete medium containing either DMSO or 10 nM BMH-21 (Selleck Chemicals) for both, non-irradiated and irradiated cells. Alternatively, SUM1315 control and SUM1315 HA-GLI1 cells were irradiated with either 1 or 2 Gy and seeded at 500 cells per well in triplicate in 6-well plates. Approximately, 10 days after seeding, plates were washed with 1×PBS and cells were fixed using 4% PFA before staining with 0.1% crystal violet (Sigma) in 10% ethanol (Pharmco) for counting. Images were taken using SMZ800 stereo zoom microscope (Nikon) and quantified using ImageJ software.

### Chromatin Immunoprecipitation Assay (ChIP)

Overall, 2 × 10^6^ cells were seeded in 10-cm culture dishes and processed using the SimpleChIP Plus Enzymatic kit (Cell Signaling Technologies, Danvers, MA) as per the manufacturer’s protocol. Briefly, cells were fixed with 1% formaldehyde at room temperature and cell pellets were processed for nuclei isolation and chromatin digestion with micrococcal nuclease and sonication. In total, 10 μg of cross-linked chromatin was immunoprecipitated with 2 μg anti-Histone H3 (acetyl K27) (Abcam, ab4729) overnight at 4 °C. Chromatin was eluted from the immunoprecipitate, and cross-links were reversed. Purified DNA from ChIP and input was subjected to RT-qPCR using 2X Maxima SYBR Green Master Mix (Thermo Fisher). To detect enrichment of H3K27ac, the following primers were used to amplify regions of the rDNA promoter Upstream -988 forward: GCTTCTCGACTCACGGTTTC, reverse: GGAGCTCTGCCTAGCTCACA, Promoter -48; forward: GAGGTATATCTTTCGCTCCGAGTC, reverse: CAGCAATAACCCGGCGG.

Threshold cycle (C[T]) values of input DNA were used to calculate the percent input of immunoprecipitation utilizing the following calculation:

Percent input = 2% × 2^(C[T] 2% Input Sample-C[T] IP Sample)^ and percent enrichment as compared to corresponding controls is depicted. Each reaction was done in triplicate using an Applied Biosystems StepOnePlus (Thermo Fisher).

### Cell proliferation assay

SUM1315 cells were seeded in a 96-well plate at a density of 7000 cells per well in either DMSO-containing media or (0.1, 0.25, 0.5, 1, 2.5 μM) M3814 (Selleck Chemicals) or (0.75, 0.38, 0.18, 0.09 μM) BMH-21 + /- 2.5 µM Vismodegib-containing media for 24 h, then irradiated with 4 Gy and incubated for 15 h. Plates then were replenished with medium containing CyQUANT Cell Proliferation Assay reagent (Thermofisher), incubated for 1 hr, and fluorescence was recorded according to manufacturers’ protocol for determination of viable cells. Readings were normalized and graphed using GraphPad Prism version 8 (GraphPad Software, La Jolla, CA). The combination index was calculated using CompuSyn software to determine whether the combination of drugs had synergistic (<1), antagonistic (>1), or no effect (=0).

### Immunofluorescence

Cells were plated on coverslips, then exposed to 4 Gy irradiation, and fixed in 3.7% formaldehyde 4 h post IR. Coverslips were then rinsed in 1× PBS before permeabilization in 1×PBS with 0.3% Triton X-100. Cells were blocked in 1X PBS with 0.3% triton X-100 and 5% BSA, then incubated overnight at 4 °C in respective primary antibodies anti-GLI1 1:250 (Cell Signaling Technologies #2643), anti-TCOF1 1:200 (Millipore Sigma #HPA038237) and anti-RPA194 1:250 (Invitrogen #PIPA556766) diluted in 1× PBS with 0.3% Triton X-100 and 1% BSA. The following day, cells were washed in 1× PBS, then incubated in respective secondary antibodies Alexa Fluor 594 goat anti-rabbit 1:400 and Alexa Fluor 488 goat anti-mouse 1:400, (Thermo Fisher) diluted in 1% BSA. For UBF1 staining, coverslips were incubated with Alexa Fluor 647-conjugated anti-UBF 1:50 (Santa Cruz, Dallas TX #sc13125) for 1 hr after the secondary antibody wash. After washing in 1× PBS, coverslips were mounted using VECTASHIELD PLUS Anti-fade Mounting Medium with DAPI (Vector Laboratories). Cells were visualized using a Nikon Eclipse Ti-U and NIS elements software was used to determine Pearson’s Correlation of intensity overlap of GLI1 and TCOF1 or RPA194 across five random fields. Representative images are depicted.

For confocal microscopy, SUM1315 cells were incubated overnight at room temperature with GLI1 (Cell Signaling Technology) and RPA194 (Invitrogen) primary antibodies in 5% BSA. After washing, cells were incubated in secondary anti-mouse and anti-rabbit IgG antibodies conjugated to Alexa Fluor 488 and 594 (Thermo Fisher) in 5% BSA, followed by three washes in 1× PBS before incubation for 1 h with TCOF1 Antibody conjugated to Alexa Fluor 647 1:250 (Santa Cruz #sc374536AF647). The cells were stained with DAPI (Fisher) and mounted with VECTASHIELD (Vector Laboratories) and analyzed using a Nikon A1R Confocal Microscope at ×60. Images were analyzed using NIS-Elements (Nikon) software.

### Immunoblotting

Cells were lysed in RIPA buffer (Millipore Sigma) containing HALT protease and phosphatase inhibitor cocktail (Thermo Fisher) and sonicated to complete lysis. Lysates were clarified by centrifugation before protein concentrations were quantified using the Precision Red assay (Cytoskeleton, Denver, CO). Equal protein was electrophoresed by SDS-PAGE and wet transferred to PVDF membranes (BioRad, Hercules, CA). When probing for proteins with molecular weights over 200 kDa, wet transfers were done at 30 V for 16 h. Primary antibodies against γ-H2AX 1:1000 (Cell Signaling Technology, # 9718S), anti-Phospho KAP-1 S824 1:200 (Bethyl lab, #A300-767A) and anti α/β-Tubulin 1:5000 (Cell Signaling Technology, 2148S) were used, as well as secondary HRP-conjugated antibodies against mouse and rabbit IgG 1:5000 (Cytivia, Marlborough, MA) when appropriate. Chemiluminescence images were captured using the Imager 600 (Amersham). Full, uncropped images are made available as Supplementary Figs. 9 and 10.

### Pulmonary metastasis assays

Pulmonary metastasis assays (PuMA) were carried out adopting the protocol published in ref. ^36^. In total, 2 × 10^5^ GFP-expressing 4T1 or 4 × 10^5^ SUM1315 cells were irradiated with 1 Gy irradiation. Four hours later the cells were injected into the tail vein of Balb/c or athymic nude mice, respectively. Fifteen minutes post injection, mice were humanely euthanized via carbon dioxide inhalation confirmed by vital tissue harvest as per the guidelines of the AVMA. Mice were placed in the chamber, then 100% CO_2_ was introduced at a fill rate of 30–70% of the chamber volume per minute with CO_2_ for a total of 3 min to induce rapid unconsciousness with minimal distress to the animals. Using sterile surgical conditions, the mice were placed in dorsal recumbency, and the sternum was removed to expose the lung and the trachea was then cannulated with 0.6% agarose in assay media (M-199 supplemented with 1.0 μg/mL crystalline bovine insulin, 0.1 μg/mL hydrocortisone, 0.1 μg/mL retinyl acetate, 100 U/mL penicillin and 100 μg/mL streptomycin, and 7.5% sodium bicarbonate) and lungs were resected. Lung sections were placed on a 2 × 2 × 0.7 cm piece of Surgifoam (Ethicon, Somerville, NJ) soaked in culture media. Culture medium was replaced with media containing 50 nM BMH-21, 20 µM Vismodegib, or a combination of both. Lung sections were flipped over with each media change twice weekly.

In a separate experiment, 2 × 10^5^ GFP-expressing 4T1 or 4 × 10^5^ SUM1315 cells were injected into the tail vein of Balb/c or athymic nude mice, respectively. Euthanasia was performed in a method consistent with the recommendations of the AVMA. Lung sections were exposed to 4 Gy irradiation and media was changed to media containing 50 nM BMH-21, 20 µM Vismodegib, or a combination of both and treated as described before. Pictures were captured using a Nikon SMZ800 stereo zoom microscope. Images were analyzed using ImageJ-FIJI software and area corrected total cell fluorescence (CTCF) was calculated as CTCF = integrated density – (area of selected cell × mean fluorescence of background readings)^37^. Animal studies were approved and conducted in accordance with University of Alabama at Birmingham Institutional Animal Care and Use Committee.

### Chromatin immunoprecipitation mass spectrometry (ChIP-MS)

SUM1315 HA-GLI1-expressing cells were exposed to 4 Gy irradiation and processed at 2 h or 4 h post irradiation, controls were collected at 4 h, then processed for ChIP. Briefly, at time of collection, cells were cross-linked using 1% formaldehyde for 10 min. Cross-linked cells were washed twice and processed for nuclei isolation and chromatin digestion with micrococcal nuclease followed by sonication. Overall, 100 µl of protein G beads were washed in 1 ml of 1× PBS + 5 mg/ml BSA for a total of four washes. In all, 3 µg of anti-RPA194 (Santa Cruz) or 3 µg of isotype was added to beads. The mixture was mixed by rotating the tube overnight at 4 °C. Antibody coupled beads were washed 4 times in 1× PBS containing 5 mg/ml BSA, and incubated with 10 μg of cross-linked chromatin overnight at 4 °C. Following incubation with chromatin, beads were washed and processed for analysis via LCMS upon processing the beads as follows: Samples were eluted in 1× final LDS sample buffer at 96° C for 10 min. The eluate was collected on a magnetic stand, reduced, and denatured further at 70 °C for 10 min. The sample was resolved using 10% Bis–tris gel and stained overnight with Colloidal Coomassie. Each sample lane was digested with trypsin overnight in six fractions prior to LCMS analysis. Data was analyzed using Scaffold 5 with the following parameters: for more stringent analysis, protein threshold was set to 99%, minimum peptides 2, and peptide threshold 80%. To reveal more hits, less stringent analysis was done with 80% protein threshold and peptide minimum set to 1. Proteins bound to isotypes controls were excluded from analysis, and quantitative value (normalized total spectra) was used to determine to fold change >1.5 in protein abundance at the rDNA with RPA194.

### Patient data analysis

Data from RNA whole-transcriptomic microarray analysis of 26 breast cancer patients’ tumors before or after irradiation were acquired from NCBI GEO (Gene Expression Omnibus) GSE65505, and used for GSEA (Gene Set Enrichment Analysis, gsea-msigdb.org). The molecular signatures database was used to examine correlations of various signatures (hallmark gene sets and curated gene sets) with pre-irradiation or post-irradiation status.

RNA sequencing data (IlluminaHiSeq) of 1247 breast cancer primary tumors from TCGA Breast Cancer was accessed from public data portal (https://xenabrowser.net) in May 2022. Data were extracted for analysis and POLR1A and TCOF1 RNA gene expression were stratified into high and low expression based on median expression and examined for overall survival. *T* test was applied for statistical analysis using GraphPad Prism (GraphPad Software, La Jolla, CA). 10-Year KM overall survival and progression-free interval values were extracted for analysis. Comparisons were considered statistically significant for *P* value < 0.05.

### Statistical analysis

Statistical analysis was conducted and graphs were plotted using GraphPad Prism software (La Jolla, CA). Student’s *t* test or ANOVA analysis was used as appropriate. Statistical significance was determined for *P*  ≤  0.05 and is denoted where applicable. Any non-significant data are denoted by “ns”. All data are represented as mean +/− SEM.

### Study approval

All animal studies were approved and conducted in accordance with the Institutional Animal Care and Use Committee (IACUC) of UAB.

### Reporting summary

Further information on research design is available in the Nature Research Reporting Summary linked to this article.

## Supplementary information

Supplementary Figures

REPORTING SUMMARY

## Data Availability

The mass spectrometry proteomics data have been deposited to the ProteomeXchange Consortium via the PRIDE partner repository with the dataset identifier PXD041472 and 10.6019/PXD041472. Data from RNA whole-transcriptomic array analysis of breast cancer patients were published by Horton et al. and were accessed from the Gene Expression Omnibus accession number GSE65505. The molecular signatures database was used to examine correlations of various signatures (hallmark gene sets and curated gene sets) with pre-irradiation or post-irradiation status. RNA sequencing data (IlluminaHiSeq) of 1247 breast cancer primary tumors from TCGA Breast Cancer was accessed from public data portal (https://xenabrowser.net) in May 2022.
