# Supplementary material for: Ribosome biosynthesis and Hedgehog activity are cooperative actionable signaling mechanisms in breast cancer following radiotherapy
Source: NPJ Precis Oncol. 2023 Jun 28;7:61. doi: 10.1038/s41698-023-00410-y (PMC10307872; doi:10.1038/s41698-023-00410-y)

Supplementary Figures

Supplementary Figure 1.

(A) Heat map represents rRNA biosynthesis signature in 4 matched breast cancer tumors pre and post IR. **(B)** GSEA of POLI regulation signature examined within tumors from 27 breast cancer patients before IR or 10 days after IR, graph is showing a trending enrichment signature in tumors post IR (NES = 0.83) **(C)** Reactome depicts top 10 gene sets of pathways enriched in tumor samples after IR in the same patient data set as in panel B, highlighting enrichment of both eukaryotic translation initiation and eukaryotic translation elongation pathways.

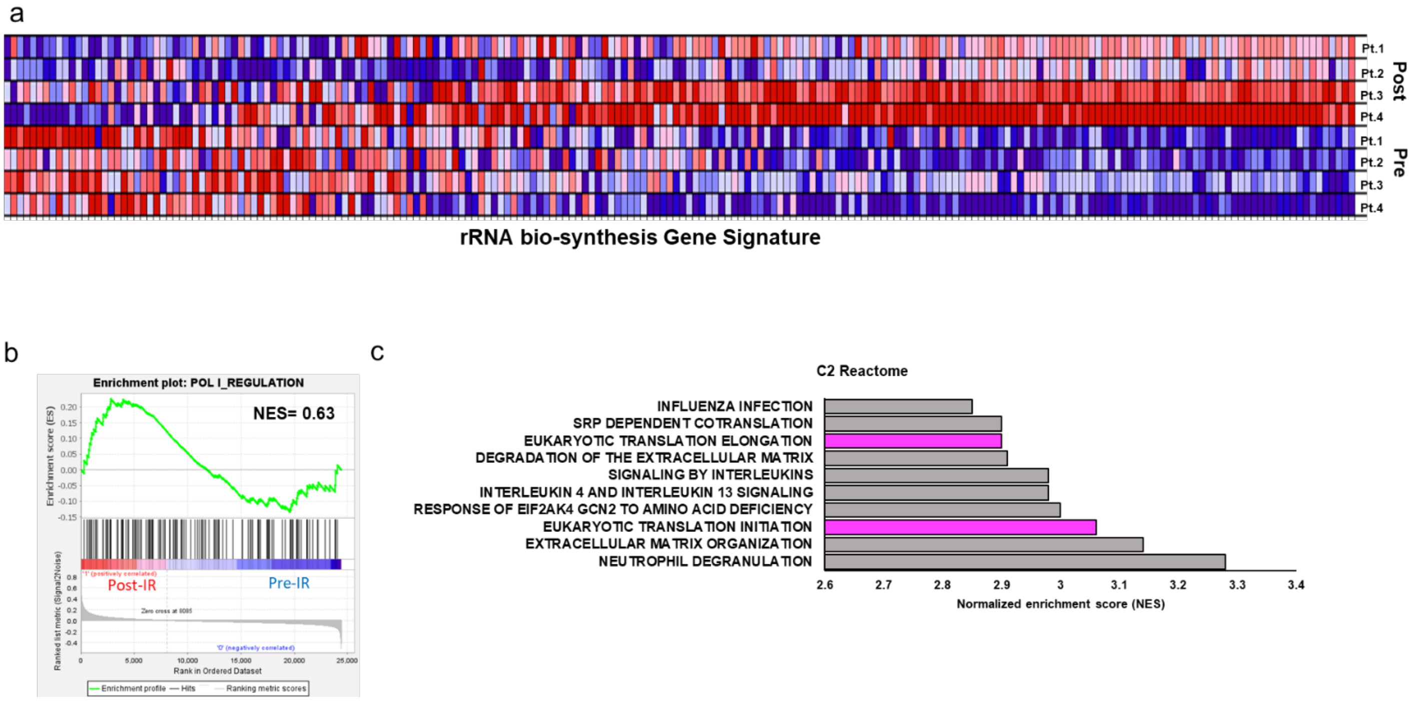

Supplementary Figure 2.

(A) Schematic of Human 45S rDNA repeating unit: ETS= External transcribed spacer; ITS= Internal Transcribed Spacer; IGS= Intergenic Sequence. Primers used for Pol activity span region of 5'-ETS from 851bp -961bp. **(B)** RT-qPCR performed using primers specific to 851-961 ETS region show increased steady state levels of rRNA, indicating increased Pol I activity after 4Gy irradiation (IR-pink) compared to control (NIR-blue) in SUM149 cells **(C)** Additional image fields of SUM1315 FUrd incorporation after 4Gy IR, FUrd (green) Fibrillarin (red). **(D)** Quantification of FUrd intensity in SUM149 after 4Gy IR FUrd (green) Fibrillarin (red). Graph indicates fold change in mean florescence intensity. 100 cells quantified in each group. **(E)** Quantification of number of nucleoli per nucleus in SUM149 cells before or after IR (4Gy). Graph represents the % of cells with 1 or 2+ nucleoli/nucleus per field. **(F)** RT-qPCR performed using primers specific to 851-961 ETS region show increased steady state levels of rRNA, indicating increased Pol I activity as early as 15 minutes to 4 hours post 4Gy irradiation (IR-pink) compared to control (NIR-blue) in SUM149. **(G)** RT-qPCR performed using primers specific to 851-961 ETS region show level of rRNA ETS transcripts unchanged, indicating no effect on Pol I activity as long as 4 hours post 4Gy irradiation (IR-pink) compared to control (NIR-blue) in HER2+ breast cancer cells (SKBr3, MDA-MB-453), Luminal breast cancer cells (T47D,MCF7) and immortalized, non-tumorigenic MCF10A cells. **(H)** Colony formation assay of SUM149 irradiated with 1Gy and seeded at 500 cells per well in triplicate. 10nM BMH-21 was administered day 1 post seeding and media changed twice weekly during the course of the assay. Foci were stained and quantified using Image J software. Representative images of one well are depicted. Bar graphs indicate mean, the error bars are the standard error of the mean, and student's t test was used for statistical comparison with p values denoted (n=3).

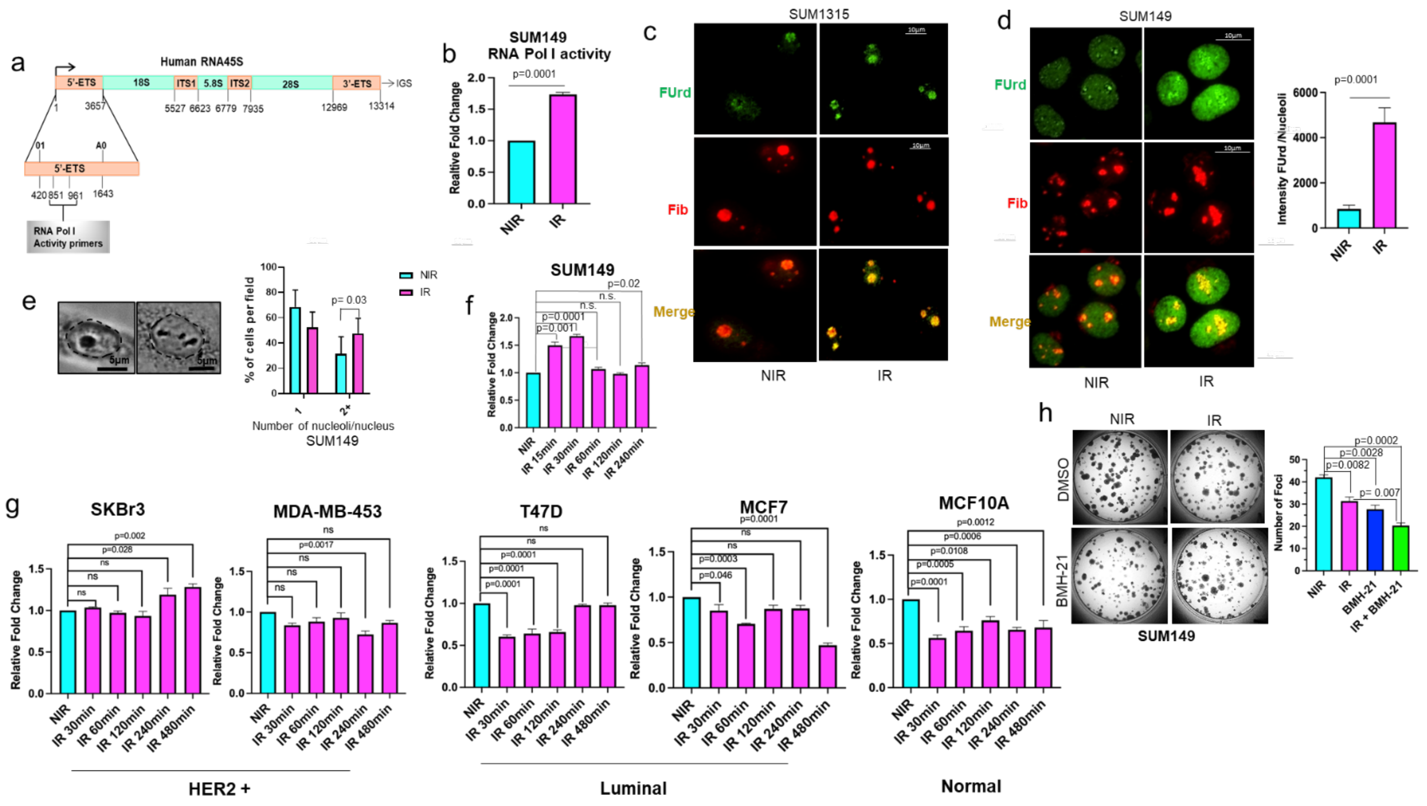

Supplementary Figure 3.

Representative images of entire lung sections from PuMA of 4T1 cells.

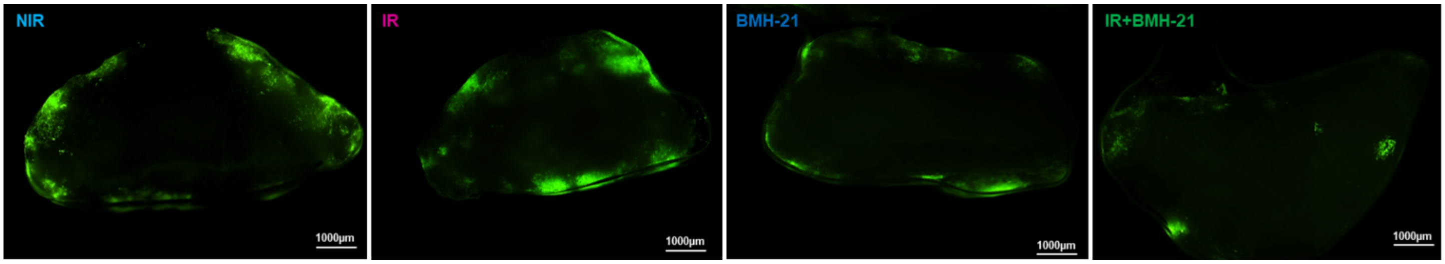

Supplementary Figure 4.

Immunofluorescence staining of MCF10A for GLI1 (green), FBL (red), and DAPI (blue) in either NIR or 1 hr post 4Gy IR conditions. Graph represents florescence intensity profile for FBL and GLI1 showing non-overlapping patterns in both IR and NIR conditions.

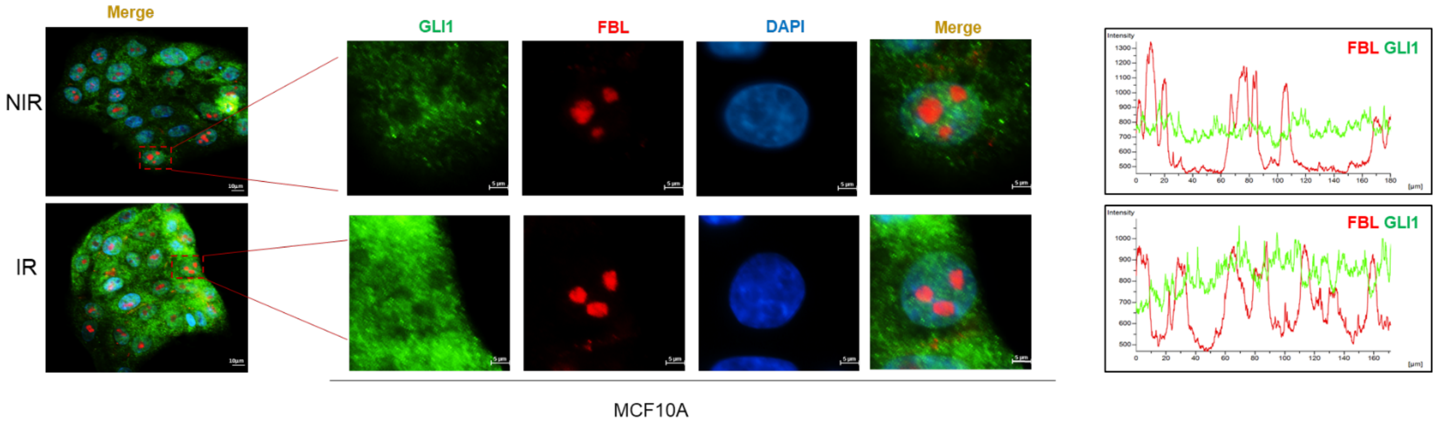

Supplementary Figure 5.

(A) Immunofluorescence staining of GLI1 (green), FBL (red), and DAPI (blue) in SUM1315 treated with cycloheximide (100 µg/ml) for one hour in NIR or 1 hr post 4Gy IR conditions. Graphs represent florescence intensity profile for FBL and GLI1 showing overlapping patterns after IR but not in NIR. **(B)** Box plots with median (center line) and maximum and minimum values (whiskers) depict Pearson correlation of FBL and GLI1 staining co-localization is significantly higher in irradiated (IR) compared to NIR conditions. Student's t test was used for statistical comparison with p values denoted.

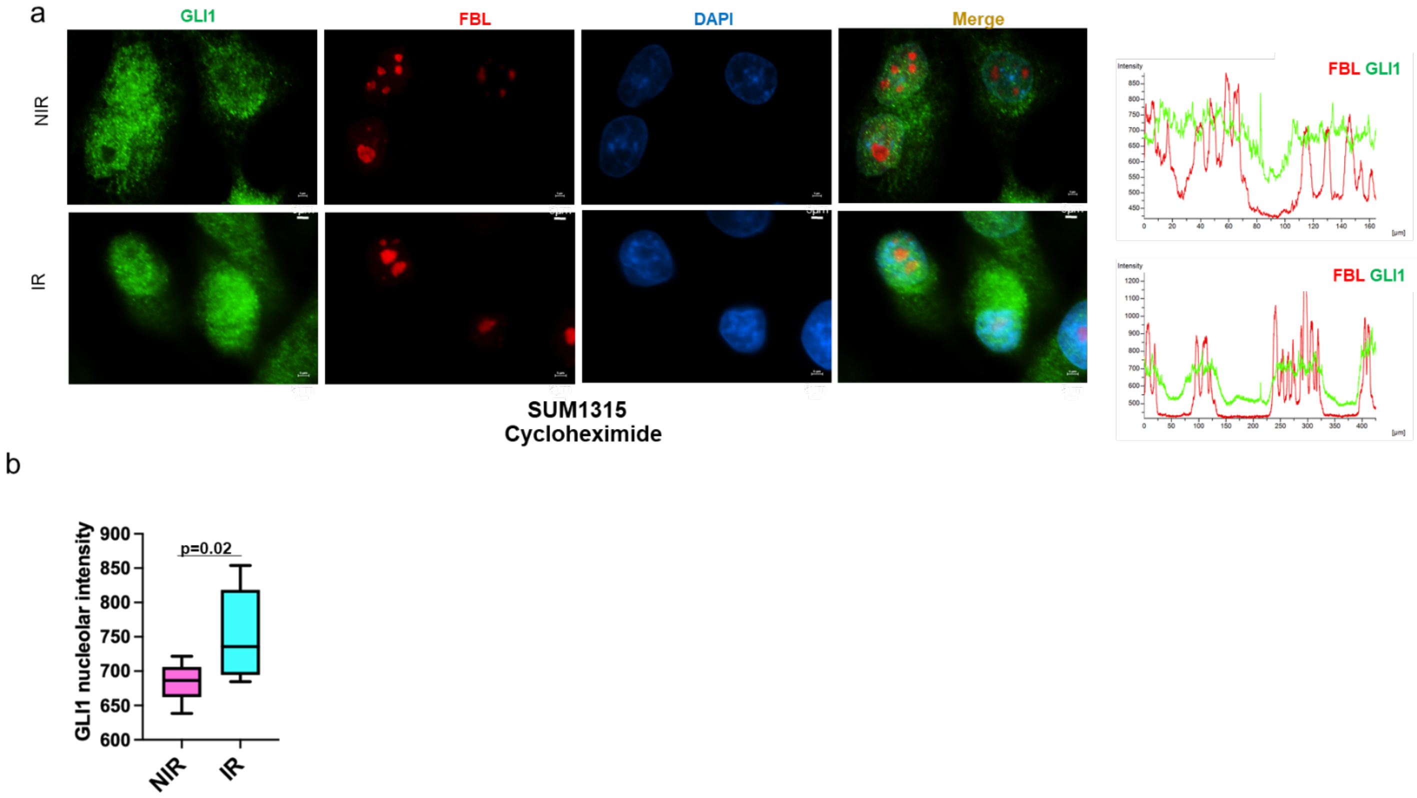

Supplementary Figure 6.

(A) Immunofluorescence staining of SUM1315 cells for GLI1 (green), POLR1A (red) & DAPI (blue) in either NIR or 4 hr post 4Gy IR conditions. **(B)** Workflow description of Chromatin Immunoprecipitation Mass Spectrometry (ChIP-MS). **(C)** Immunofluorescence staining of GLI1 (green), TCOF1 (red), DAPI (blue). Graphs represent intensity overlay of GLI1 and TCOF in control or 4 hours post 4Gy irradiation. **(D)** Box plots with median (center line) and maximum and minimum values (whiskers) depict Pearson's correlation of GLI1-TCOF1 staining in NIR and IR. Student's t test was used for statistical comparison with p values denoted. **(E)** 2D confocal immunofluorescence of GLI1 (green), TCOF1 (blue), POLR1A (red), DAPI (gray) localization 4 hours post 4Gy irradiation. **(F)** Plots demonstrate regions of intensity overlay between GLI1, POLR1A, and TCOF1 following 4Gy IR. **(G)** Decreased levels of TCOF1 transcript in SUM1315 TCOF1 silenced cells confirms knockdown. **(H)** TCOF1-silencing in SUM1315 cells hinders nucleolar translocation of GLI1. Bar graphs indicate mean, the error bars are the standard error of the mean, and student's t test was used for statistical comparison with p values denoted (n=3).

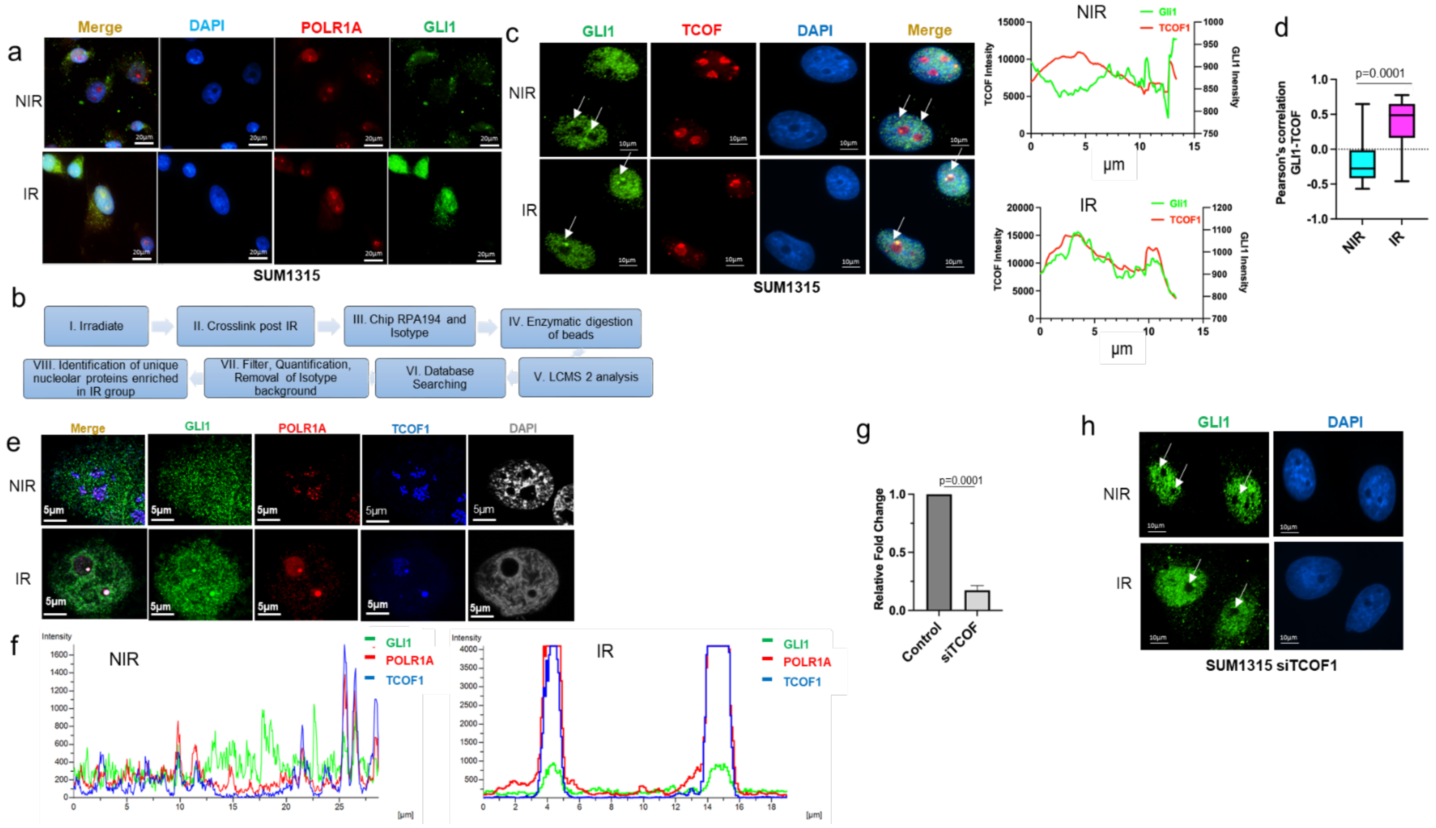

Supplementary Figure 7.

(A) Schematic of treatment with DNA-PK inhibitor (NU7441) or ATM inhibitor (KU5593) followed by irradiation (4 Gy) and harvesting of RNA at times indicated. **(B)** Immunoblotting of γH2AX in SUM1315 cells treated with either vehicle control or DNAPKcs i at either 2.5, 5 or 10 μM concentrations for 1 hr before either NIR or IR (4 Gy) at different time points of 30, 60, 240 and 480 minutes. Full length blots in supplemental figure 9. **(C)** Immunoblotting of p-KAP1 in SUM1315 cells treated with either vehicle control or ATM i at either 5, 10 or 20 μM concentrations for 1 hr before either NIR or IR (4 Gy) at different time points of 30, 60, 240 and 480 minutes. Full length blots in supplemental figure 10. The ability of SUM149 cells to activate RNA Pol I is significantly reduced when treated with **(D)** 2.5 μM DNAPKc I or **(E)** 5 μM ATM i. SUM149 HA-GLI1 cells demonstrate upregulated Pol I activity following irradiation that remains unaffected when treated with **(F)** 2.5 μM DNAPKc i or **(G)** 5 μM ATM i. **(H)** Determination of CI (combination index) of Vismodegib and M3814. Percent viability of SUM1315 cells determined using various concentration of M3814 (alone, red curve) or in combination with 2.5 μM Vismodegib (blue curve) in addition to 4 Gy IR. The adjacent table shows the determination of CI for each combination. Bar graphs indicate mean, the error bars are the standard error of the mean, and student's t test was used for statistical comparison with p values denoted (n=3).

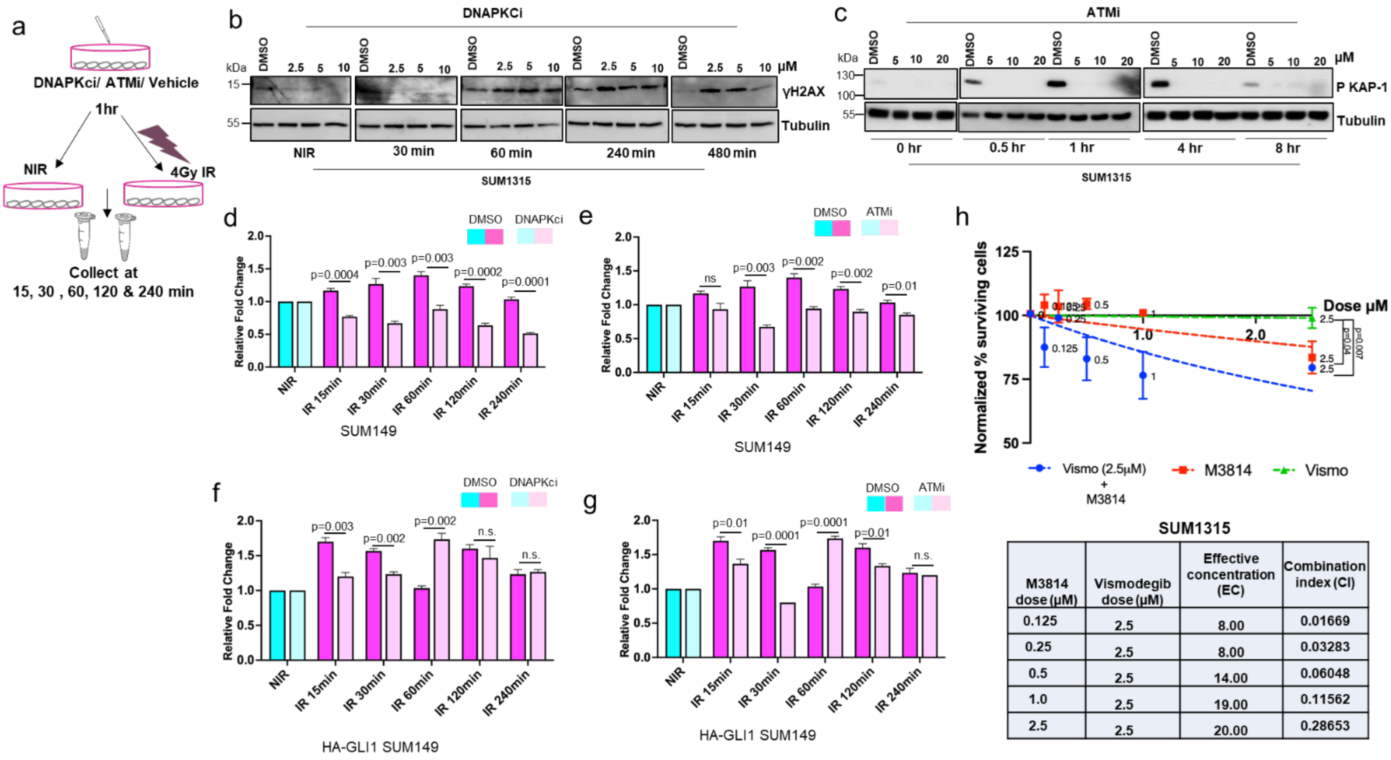

SUM1315

M3814 dose (μM)	Vismodegib dose (μM)	Effective concentration (EC)	Combination index (CI)
0.125	2.5	8.00	0.01669
0.25	2.5	8.00	0.03283
0.5	2.5	14.00	0.06048
1.0	2.5	19.00	0.11562
2.5	2.5	20.00	0.28653

Supplementary Figure 8.

(A) Determination of CI (combination index) of Vismodegib and BHM-21. Percent viability of SUM1315 cells is determined using various concentration of BMH-21 (alone, red curve) or in combination with 2.5 μM Vismodegib (blue curve) in addition to 4 Gy IR. Line graphs indicate mean, the error bars are the standard error of the mean, and student's t test was used for statistical comparison with p values denoted (n=3). The adjacent table shows the determination of CI for each combination. **(B)** PuMA of SUM1315 cells, wherein lung sections were irradiated then treated with BHM-21 and Vismodegib. Box plots with median (center line) and maximum and minimum values (whiskers) depict relative total corrected fluorescence analyzed from six sections per group. Student's t test was used for statistical comparison with p values denoted (n=6).

a

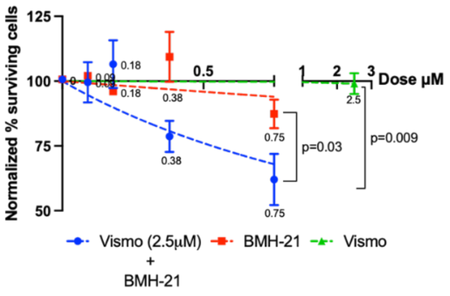

BMH-21 dose (µM)	Vismodegib dose (µM)	Effective concentration (EC)	Combination index (CI)
0.75	2.5	48.00	0.41488
0.38	2.5	22.00	0.23099
0.18	2.5	6.00	0.12355
0.09	2.5	0	238975.

b

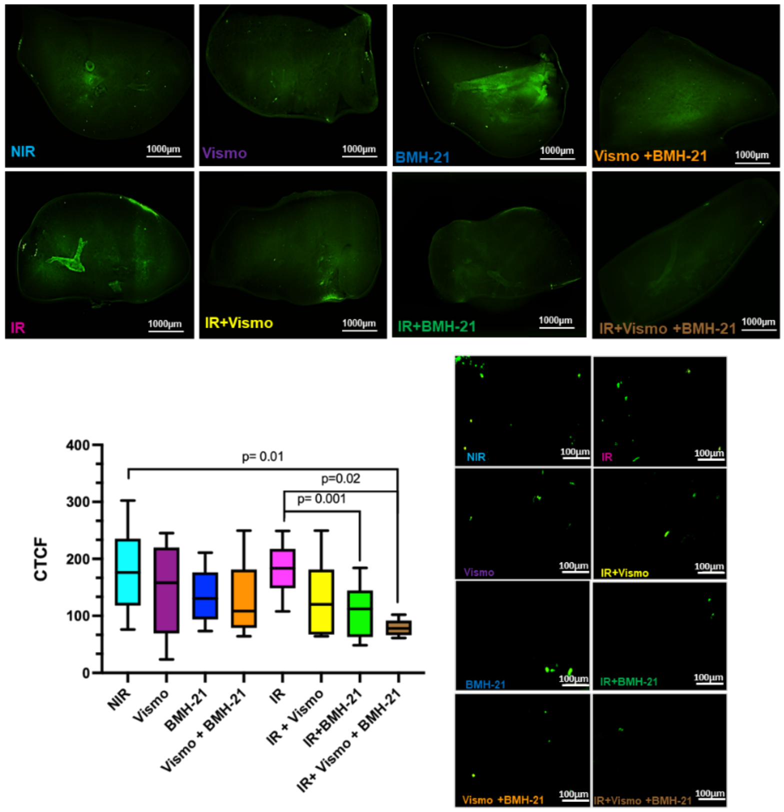

Supplementary Figure 9.

Full length uncropped immunoblots of γH2AX and tubulin presented in supplemental figure 7b.

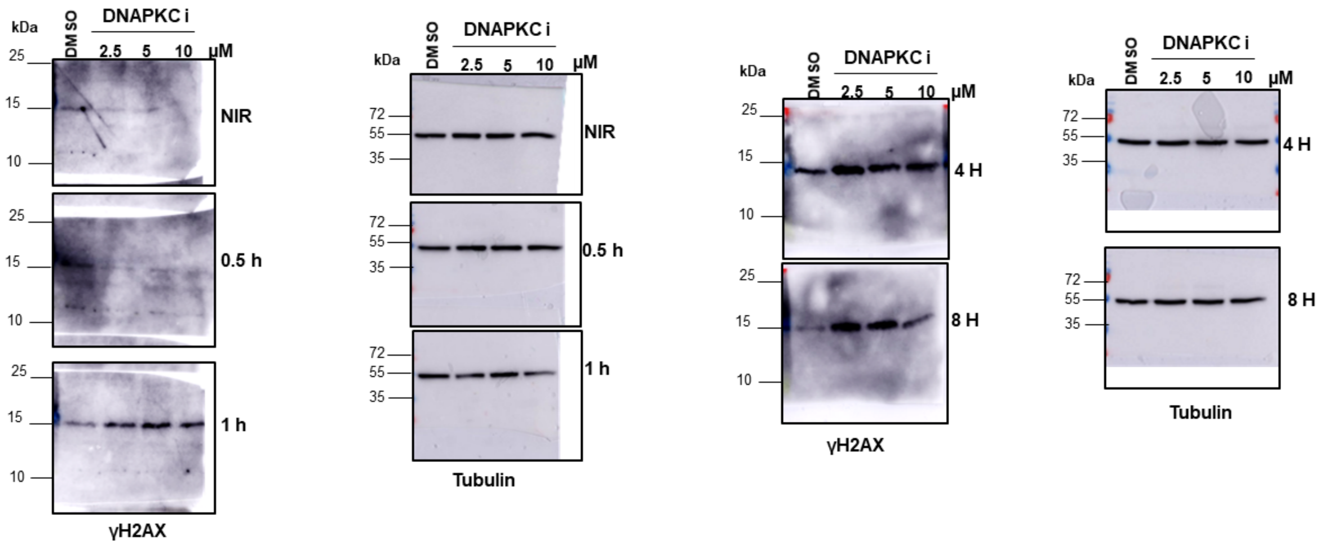

Supplementary Figure 10.

Full length uncropped immunoblots of p-KAP1 and tubulin presented in supplemental figure 7c.

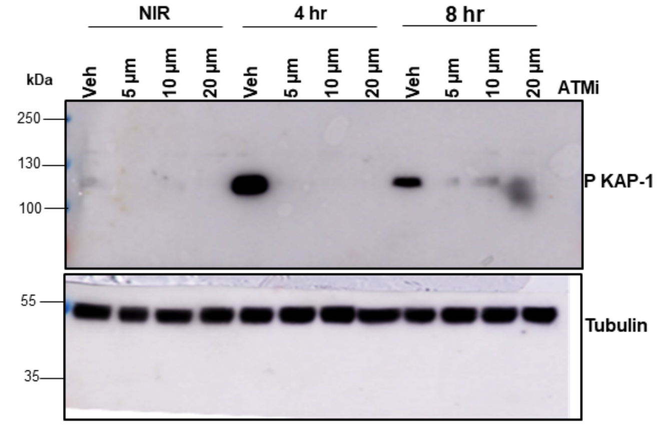

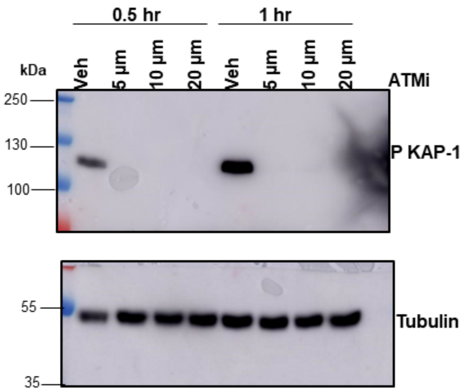

Supplement: Supplementary file 1 — Supplementary Figures [file 41698_2023_410_MOESM1_ESM.pdf]
