# Supplementary material for: Ribosome biosynthesis and Hedgehog activity are cooperative actionable signaling mechanisms in breast cancer following radiotherapy
Source: NPJ Precis Oncol. 2023 Jun 28;7:61. doi: 10.1038/s41698-023-00410-y (PMC10307872; doi:10.1038/s41698-023-00410-y)
Supplement: Supplementary file 2 — REPORTING SUMMARY [file 41698_2023_410_MOESM2_ESM.pdf]

nature portfolio

Corresponding author(s): Professor Lalita Shevde-Samant

Last updated by author(s): May 24, 2023

Reporting Summary

Nature Portfolio wishes to improve the reproducibility of the work that we publish. This form provides structure for consistency and transparency in reporting. For further information on Nature Portfolio policies, see our Editorial Policies and the Editorial Policy Checklist.

Statistics

For all statistical analyses, confirm that the following items are present in the figure legend, table legend, main text, or Methods section.

n/a	Confirmed	
☐	☒	The exact sample size (*n*) for each experimental group/condition, given as a discrete number and unit of measurement
☐	☒	A statement on whether measurements were taken from distinct samples or whether the same sample was measured repeatedly
☐	☒	The statistical test(s) used AND whether they are one- or two-sided<br>*Only common tests should be described solely by name; describe more complex techniques in the Methods section.*
☒	☐	A description of all covariates tested
☒	☐	A description of any assumptions or corrections, such as tests of normality and adjustment for multiple comparisons
☐	☒	A full description of the statistical parameters including central tendency (e.g. means) or other basic estimates (e.g. regression coefficient) AND variation (e.g. standard deviation) or associated estimates of uncertainty (e.g. confidence intervals)
☐	☒	For null hypothesis testing, the test statistic (e.g. *F*, *t*, *r*) with confidence intervals, effect sizes, degrees of freedom and *P* value noted<br>*Give P values as exact values whenever suitable.*
☒	☐	For Bayesian analysis, information on the choice of priors and Markov chain Monte Carlo settings
☒	☐	For hierarchical and complex designs, identification of the appropriate level for tests and full reporting of outcomes
☒	☐	Estimates of effect sizes (e.g. Cohen's *d*, Pearson's *r*), indicating how they were calculated

Our web collection on statistics for biologists contains articles on many of the points above.

Software and code

Policy information about availability of computer code

Data collection	No software was used for data collection.
Data analysis	Data analysis, statistical testing, and visualizations were performed using Graphpad Prism (v9.5.1). Analysis and capturing of all immunofluorescence and imaging data was done using NIS Elements AR (v5.2.02). Gene Set Enrichment Analysis was performed using GSEA software (Broad Institute, v4.2.3). Mass Spectrometry data was analyzed using Scaffold 5, Proteome Software Inc. (v5.01)

For manuscripts utilizing custom algorithms or software that are central to the research but not yet described in published literature, software must be made available to editors and reviewers. We strongly encourage code deposition in a community repository (e.g. GitHub). See the Nature Portfolio guidelines for submitting code & software for further information.

Data

Policy information about availability of data

All manuscripts must include a data availability statement. This statement should provide the following information, where applicable:

- Accession codes, unique identifiers, or web links for publicly available datasets
- A description of any restrictions on data availability
- For clinical datasets or third party data, please ensure that the statement adheres to our policy

Mass Spectrometry data has been deposited with the PRIDE partner repository under the identifier PXD041472. Data from RNA whole transcriptomic array analysis

of breast cancer patients were published by Horton et al. and were accessed from the Gene Expression Omnibus accession number GSE65505. TCGA Breast tumor data was acquired from Xenabrowser (UCSC).

Research involving human participants, their data, or biological material

Policy information about studies with human participants or human data. See also policy information about sex, gender (identity/presentation), and sexual orientation and race, ethnicity and racism.

Reporting on sex and gender	N/A
Reporting on race, ethnicity, or other socially relevant groupings	N/A
Population characteristics	N/A
Recruitment	N/A
Ethics oversight	N/A

Note that full information on the approval of the study protocol must also be provided in the manuscript.

Field-specific reporting

Please select the one below that is the best fit for your research. If you are not sure, read the appropriate sections before making your selection.

☒ Life sciences ☐ Behavioural & social sciences ☐ Ecological, evolutionary & environmental sciences

For a reference copy of the document with all sections, see nature.com/documents/nr-reporting-summary-flat.pdf

Life sciences study design

All studies must disclose on these points even when the disclosure is negative.

Sample size	All results are representative of experimental findings from experimental replicates of at least an n=3. All results were replicated using at least two model systems as indicated. All data is represented as mean +/- SEM. Statistical comparisons analyzed in Graphpad Prism using either student's T test or multiple comparison test as denoted in figures with p values.
Data exclusions	No data were excluded.
Replication	All data was reproduced across multiple experiments and cell line model systems.
Randomization	N/A
Blinding	During experimental procedures investigators were not blinded.

Reporting for specific materials, systems and methods

We require information from authors about some types of materials, experimental systems and methods used in many studies. Here, indicate whether each material, system or method listed is relevant to your study. If you are not sure if a list item applies to your research, read the appropriate section before selecting a response.

Materials & experimental systems

n/a	Involved in the study	
☐	☒	Antibodies
☐	☒	Eukaryotic cell lines
☒	☐	Palaeontology and archaeology
☐	☒	Animals and other organisms
☒	☐	Clinical data
☒	☐	Dual use research of concern
☒	☐	Plants

Methods

n/a	Involved in the study	
☒	☐	ChIP-seq
☒	☐	Flow cytometry
☒	☐	MRI-based neuroimaging

Antibodies

Antibodies used	anti-Brdu Sigma cat#B8434, anti-Fibrillarin Abcam cat#ab166630, anti-Histone H3 acetyl K27 Abcam cat#ab4729, anti-GLI1 Cell Signaling Technology cat#2643S, anti-TCOF1 Millipore Sigma cat#HPA038237, anti-RPA194 Invitrogen cat#PIPA556766, anti-Treacle AlexaFluro647 Santa Cruz cat#sc-374536AF647,anti-UBTF AlexFluor 647 Santa Cruz #sc13125, anti- gamma H2AX Cell Signaling Technology cat#9718S, anti-phospho KAP-1 S824 Bethyl Lab cat#A300-767A, anti α/β-Tubulin Cell Signaling Technology cat# 2148S The dilutions at which each antibody was used is specified in the manuscript (methods section).
Validation	All antibodies used were validated by manufacturer, used as per recommended protocols, and tested for each application using appropriate controls.

Eukaryotic cell lines

Policy information about cell lines and Sex and Gender in Research

Cell line source(s)	Most cell lines were obtained from ATCC and grown according to standard culture conditions., SUM1315 and SUM149 were obtained from Asterand Biotech and cultured according to standard culture conditions.
Authentication	Cell lines were authenticated using ATCC STR analysis.
Mycoplasma contamination	Cell lines were mycoplasma tested using PlasmoTest (InvivoGen).
Commonly misidentified lines (See ICLAC register)	None of these lines are listed in this database.

Animals and other research organisms

Policy information about studies involving animals; ARRIVE guidelines recommended for reporting animal research, and Sex and Gender in Research

Laboratory animals	6 week old Balb/c female mice were purchased from Jackson Laboratory
Wild animals	No wild animals were used in these studies.
Reporting on sex	Only female mice were used as studies required formation of mammary tumors, and for overall study relevance.
Field-collected samples	N/A
Ethics oversight	All anmial studies were conducted in accordance with the UAB-IACUC.

Note that full information on the approval of the study protocol must also be provided in the manuscript.
